# PRELID1 and VDAC3 Coordinate a Senescence‐Like State in Germinal Center B Cells to Promote IL‐7–Driven Antitumor Immunity in Colorectal Cancer

**DOI:** 10.1002/advs.202521951

**Published:** 2026-02-18

**Authors:** Yuhan Liao, Huimeng Xu, Xinghua Zhuo, Shupeng Hu, Lanhui Huang, Zhe Hao, Jianxiong Chen, Yulu Wang, Jun Zhou

**Affiliations:** ^1^ Department of Pathology Nanfang Hospital School of Basic Medical Sciences Southern Medical University Guangzhou Guangdong China; ^2^ Guangdong Province Key Laboratory of Molecular Tumor Pathology Guangzhou Guangdong China; ^3^ School of Computer Science Guangdong Polytechnic Normal University Guangzhou China; ^4^ School of Computer Science University of Manchester Manchester UK; ^5^ Department of Pathology The Tenth Affiliated Hospital (Dongguan People's Hospital) Southern Medical University Dongguan China; ^6^ Dongguan Clinical Pathology Diagnosis Center Dongguan China; ^7^ Dongguan Key Laboratory of Clinical Pathology Dongguan China

**Keywords:** cancer microenvironment, cellular senescence, colorectal cancer, immune checkpoint blockade, mitochondria‐lysosome interactions

## Abstract

Colorectal cancer (CRC) is characterized by an immune‐suppressive microenvironment that facilitates to tumor progression and immunotherapy resistance. Emerging evidence indicates that tumor‐infiltrating B cell subsets play dual roles in modulating antitumor immunity. However, the mechanisms underlying their regulatory functions remain poorly understood. In this study, we constructed single‐cell transcriptomic analyses and a Bgc adoptive transfer model in B cell‐deficient mice, performing multifaceted validation to confirm that targeting the PRELID1–VDAC3–IL‐7 axis in senescence‐like germinal center B cells enhances sensitivity to anti‐PD‐L1 immunotherapy. Mechanistically, we identify a senescence‐like state in germinal center B cells (Bgc) that enhances antitumor immunity in CRC by promoting Interleukin‐7 (IL‐7) secretion and alleviating CD8^+^ T cell exhaustion. Specifically, this senescence‐like state is driven by PRELID1 and VDAC3 through their cooperative regulation of mitochondria‐lysosome interactions in Bgc cells, which enhances IL‐7 secretion and promotes functional crosstalk with CD8^+^ T cells to sustain antitumor immunity. In summary, our findings support a combinatorial strategy integrating targeting of the PRELID1–VDAC3–IL‐7 axis in senescence‐like Bgc cells with anti‐PD‐L1 immunotherapy for colorectal cancer.

## Graphical abstract

Colorectal cancer is characterized by an immunosuppressive microenvironment that limits immunotherapy efficacy. We identify a senescence‐like state in germinal center B cells driven by the PRELID1–VDAC3 axis, which promotes IL‐7 secretion, alleviates CD8^+^ T‐cell exhaustion, and enhances antitumor immunity. Targeting this pathway improves responsiveness to anti–PD‐L1 therapy, supporting a combinatorial immunotherapeutic strategy.

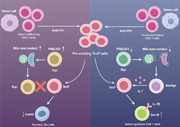

## 1 Introduction

Colorectal cancer (CRC) is one of the most life‐threatening malignancies worldwide. Among all types of cancers, CRC ranks third in incidence globally [1]. In recent years, immune checkpoint blockade (ICB) therapy has significantly enhanced clinical outcomes across various cancers [2, 3], such as melanoma, breast cancer, non‐small cell lung cancer, and gastrointestinal tumors [4, 5, 6, 7]. Despite its success, ICB therapy is currently limited to patients with DNA mismatch repair deficiency (dMMR) or high microsatellite instability (MSI‐H) in metastatic colorectal cancer (mCRC), which accounts for only about 15% of all CRC cases [8]. A key challenge in current research is to extend the therapeutic benefits of immunotherapy to a wider population of CRC patients.

Dysregulated immune responses within the tumor microenvironment (TME) are often associated with the accumulation of exhausted T cells (Tex cells), which, despite remaining viable, exhibit diminished effector functions [9]. Tex cells are characterized by the upregulated expression of multiple co‐inhibitory receptors, among which PD‐1 is the most prominent, along with others such as LAG3, TIM3, and TIGIT. B cells within the TME consist of heterogeneous subpopulations, including naïve B cells (Bn), memory B cells (Bmem), follicular B cells (Bfoc), and germinal center B cells (Bgc) [10, 11]. In addition to their pro‐tumorigenic functions [12, 13], B cells also exhibit anti‐tumor activity [14].

Mitochondria and lysosomes are two essential organelles responsible for maintaining cellular homeostasis [15, 16]. Their functions are closely interconnected, and dysfunction of those organelles is strongly associated with cellular senescence [17]. In recent years, increasing evidence has shown that mitochondria and lysosomes communicate through mitochondria‐lysosome contact sites [18, 19, 20, 21]. However, the mechanisms underlying the coordinated regulation of mitochondrial and lysosomal homeostasis remain unclear.

PRELI domain‐containing protein 1 (PRELID1) is a mitochondrial intermembrane space protein that mediates the transport of phosphatidic acid (PA) from the outer mitochondrial membrane (OMM) to the inner mitochondrial membrane (IMM) [22, 23]. Voltage‐Dependent Anion Channels (VDACs) are channel proteins located on the outer mitochondrial membrane that mediate the exchange of metabolites between the cytosol and the mitochondria. Recent studies have shown that mitochondrial VDACs and lysosomal Mucolipin‐1 (TRPML1) mediate mitochondria–lysosome interactions [18]. The maintenance of mitochondrial and lysosomal homeostasis plays a crucial role in counteracting cellular senescence [17].

Deciphering the mechanisms through which B cell subsets modulate the tumor immune microenvironment remains difficult. In this study, a senescence‐like state in Bgc that enhances antitumor immunity in CRC by promoting Interleukin‐7 (IL‐7) secretion and mitigating CD8^+^ T cell exhaustion. Studies have highlighted the potential of IL‐7 to enhance antitumor immunity and synergize with immune checkpoint blockade [24]. This functional state is regulated by PRELID1 and VDAC3 through their coordination of mitochondria–lysosome interactions within Bgc cells. To our knowledge, this is the first study to report the role of Bgc cells in antitumor immunity in CRC and elucidate the underlying mechanisms.

## 2 Results

### 2.1 Bgc Cells Promote Antitumor Immunity via Crosstalk with CD8^+^ T Cells

**FIGURE 1:**
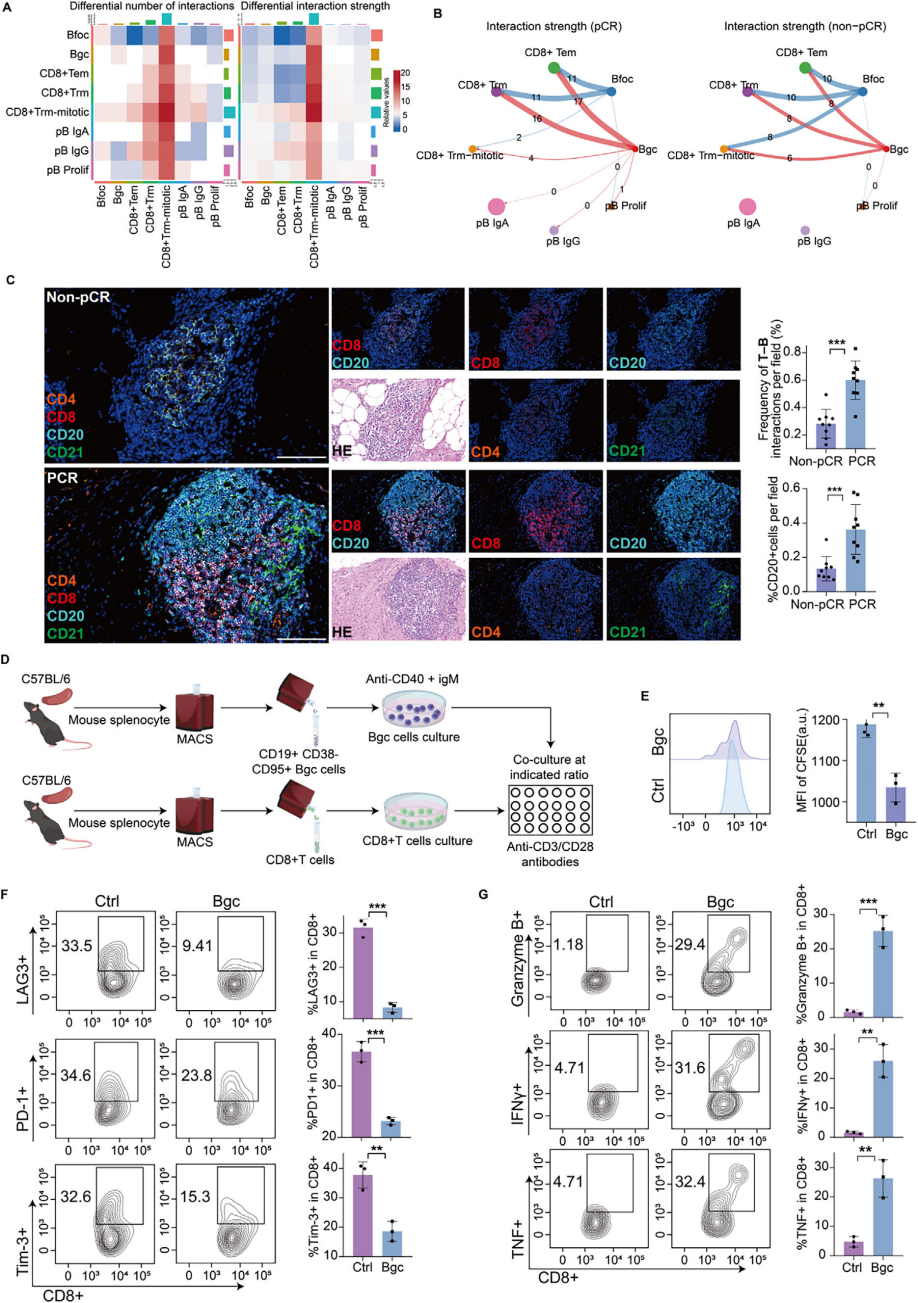
Bgc cells promote CD8^+^ T cell effector responses in ICI‐sensitive colorectal tumors. (A) Heatmap depicting inferred intercellular communication strength between B cells and CD8^+^ T cells in pCR and non‐pCR groups, where colors from deep blue to white to dark red represent increasing communication scores. (B) Quantitative analysis of cell–cell interaction strength in pCR and non‐pCR samples, with edges representing cell‐cell communication and line width corresponding to relative interaction intensity. (C) Representative multiplex immunofluorescence (mIF) images showing CD8^+^ T cells (red), CD20^+^ B cells (cyan), CD4^+^ T cells (orange), and CD21^+^ cells (green) in pCR and non‐pCR CRC tissue sections. Quantification of the merged area between CD20^+^ and CD8^+^ signals is shown on the right. Quantification of B‐cell proportions and T‐B interaction frequencies (merged area between CD20^+^ and CD8^+^ signals, defined as CD20^+^ and CD8^+^ cells within a predefined interaction radius of 20 µm) is shown on the right (*n* = 9 per group, with statistical comparisons performed using an unpaired two‐sided Student's *t*‐test). Scale bars, 50 µm. (D) Schematic of the experimental setup for co‐culture of CD8^+^ T cells with Bgc cells. (E) Carboxyfluorescein succinimidyl ester (CFSE) dilution assay showing proliferation of CD8^+^ T cells in co‐culture with Bgc cells. Data represent *n* = 3 independent experiments per group and are presented as mean ± SEM, with statistical analysis performed using unpaired two‐sided Student's *t*‐test. (F,G) Flow cytometry analysis was performed to assess the expression of exhaustion markers (PD‐1, LAG3, and Tim‐3) and cytotoxic molecules (Granzyme B, IFN‐γ, and TNF) in CD8^+^ T cells. Results shown are from *n* = 3 independent experiments per group, with statistical comparisons performed using an unpaired two‐sided Student's *t*‐test. Data are presented as mean ± SEM; ^*^ *p*<0.05, ^**^ *p*<0.01, ^***^ *p*<0.001, ns not significant.

**FIGURE 2:**
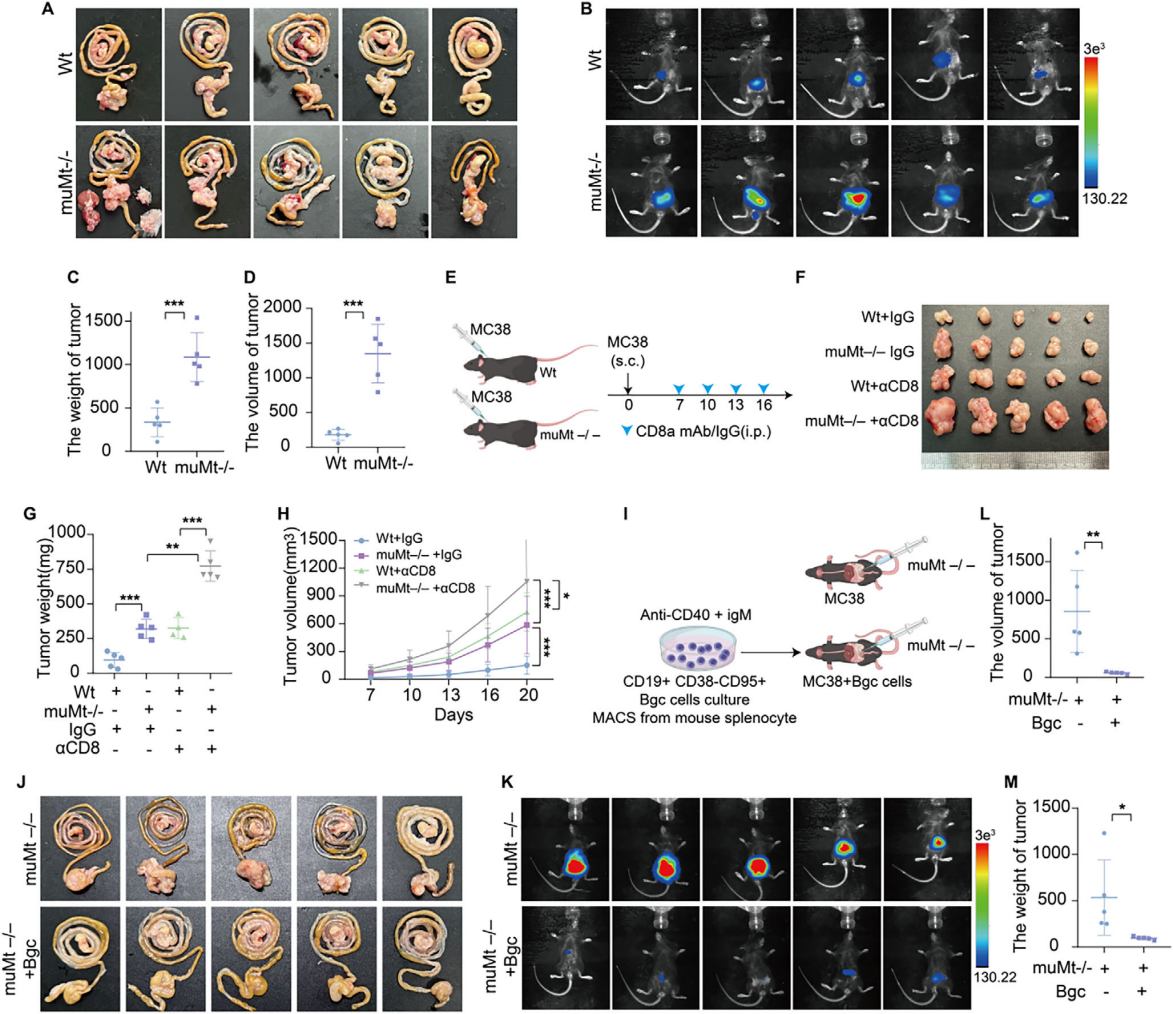
Bgc cells enhance antitumor immunity in vivo and inhibit tumor progression in B cell‐deficient mice (muMt^−/−^). (A) Representative gross images of cecal tumors from wild‐type and muMt^−/−^ mice in the in situ colorectal cancer model. (B) Representative bioluminescence imaging of tumor burden in wild‐type and muMt^−/−^ mice. (C) Tumor weights measured on the day of sacrifice (*n* = 5 per group, with statistical comparisons performed using an unpaired two‐sided Student's *t*‐test). (D) Tumor volumes measured on the day of sacrifice (*n* = 5 per group, with statistical comparisons performed using an unpaired two‐sided Student's *t*‐test). (E) Schematic diagram of the subcutaneous tumor model showing four treatment groups: Wt+IgG, muM^−/−^ +IgG, Wt +αCD8, and muMt^−/−^ +αCD8. (F) Representative images of subcutaneous tumors from each treatment group at the experimental endpoint. (G) Tumor weights measured on the day of sacrifice (*n* = 5 per group). Statistical significance was determined using one‐way ANOVA with post hoc tests. (H) Tumor volume growth curves over time (*n* = 5 per group). Statistical significance was determined using two‐way repeated‐measures ANOVA. (I) Schematic illustration of the adoptive transfer of Bgc cells into muMt^−/−^ mice in an in situ colorectal cancer model. (J) Representative gross images of cecal tumors from muMt^−/−^ mice with or without Bgc cell transfer. (K) Representative bioluminescence imaging showing tumor burden in each group. (L) Tumor volumes measured on the day of sacrifice (*n* = 5 per group, with statistical comparisons performed using an unpaired two‐sided Student's *t*‐test). (M) Tumor weights were measured on the day of sacrifice (*n* = 5 per group, with statistical comparisons performed using an unpaired two‐sided Student's *t*‐test). I.p., intraperitoneal injection. Data are presented as mean ± SEM; ^*^ *p*<0.05, ^**^ *p*<0.01, ^***^ *p*<0.001, ns not significant.

Using publicly available single‐cell RNA sequencing (scRNA‐seq) data GSE205506 from CRC tissues treated with immune checkpoint inhibitors (ICIs), we performed CellChat analysis to compare pathological complete response (pCR) and non‐pCR groups. We found that Bgc cell‐to‐CD8^+^ T cell communication was markedly enhanced in the pCR group compared to the non‐pCR group (Figure 1A,B; Figure S1). This analysis served as a discovery step, identifying Bgc cells as a candidate cellular predictor of response to immune checkpoint blockade. This observation was further validated by multiplex immunofluorescence (mIF), which revealed increased B‐T cell interactions in the pCR group (Figure 1C). To functionally validate this discovery, we performed a series of in vitro and in vivo experiments to directly assess the role and specificity of Bgc cells in modulating CD8^+^ T cell function and antitumor immunity. To investigate the crosstalk between Bgc cells and CD8^+^ T cells, we isolated CD19^+^CD95^+^CD38^−^ Bgc [14, 25] cells by magnetic activated cell sorting (MACS) from mouse spleen and co‐cultured them with activated CD8^+^ T cells at a 1:2 ratio for 48 h (Figures S2 and S3A). Flow cytometry analysis revealed that co‐culture with Bgc cells reduced the expression of exhaustion markers and enhanced both the effector function and proliferative capacity of CD8^+^ T cells compared to the control group (Figure 1D–G). To determine whether this effect was specific to Bgc cells, CD8^+^ T cells were co‐cultured with naïve, memory, or follicular (FO) B cells, followed by flow‐cytometric assessment of exhaustion marker expression. The expression levels of exhaustion markers in these co‐culture conditions remained comparable to those in the control group (Figures S3B and S4). In an in situ tumor model, tumor growth was accelerated in B cell‐deficient mice (muMt^−/−^) compared to wild‐type mice (Figure 2A–D). This conclusion was further supported by the subcutaneous tumor model, in which CD8^+^ T cell depletion resulted in a more pronounced increase in tumor proliferation (Figure 2E–H). Furthermore, adoptive transfer of primary mouse Bgc cells into muMt^−/−^ mice significantly slowed tumor growth (Figure 2I–M). To verify the homing and intratumoral localization of adoptively transferred Bgc cells, we performed a flow cytometry‐based tracking experiment using congenic donor–recipient mice. Bgc cells isolated from B6.SJL (CD45.1^+^) donors were intravenously transferred into tumor‐bearing C57BL/6J or MuMt^−/−^ recipients (CD45.2^+^), with control mice receiving no adoptive transfer [26]. In control tumors, CD45.1^+^CD19^+^ cells were nearly undetectable, confirming minimal background signal. In contrast, tumors from the adoptive transfer group contained a readily detectable population of CD45.1^+^CD19^+^ cells, accounting for more than 10% of total CD19^+^ B cells. Notably, a substantial fraction of these donor‐derived cells exhibited a CD19^+^CD95^+^IgD^−^ phenotype, consistent with the characteristic features of Bgc cells (Figure S14A,B). Flow cytometric analysis of tumor‐infiltrating lymphocytes also showed that CD8^+^ T cells from muMt^−/−^ mice receiving adoptive transfer of Bgc cells exhibited higher levels of effector function markers compared to those from muMt^−/−^ mice without adoptive transfer (Figure S5). To determine whether this effect was specific to Bgc cells, adoptive transfer of naïve B cells, memory B cells, or FO B cells was performed in the orthotopic colorectal cancer model, and tumor growth was monitored over time. Tumor growth in mice receiving these other B‐cell subsets remained comparable to control mice (Figure S6).

### 2.2 PRELID1 and VDAC3 Cooperatively Regulate Bgc Cell Activation, Leading to Enhanced Antitumor Immunity

**FIGURE 3:**
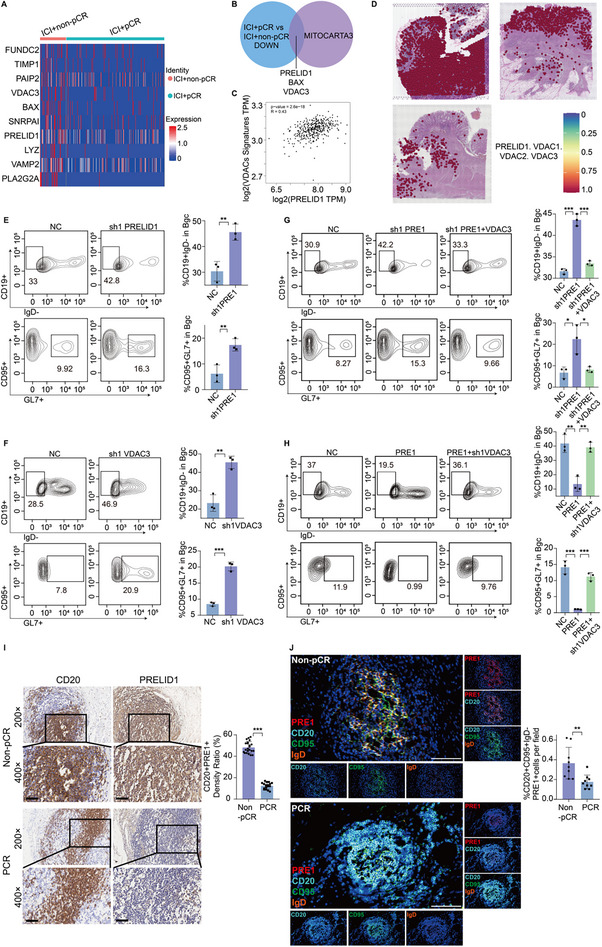
PRELID1 and VDAC3 cooperatively regulate Bgc cell activation. (A) Differential gene expression analysis was performed to identify the top ten downregulated proteins of Bgc cells in the pCR group based on log fold change (logFC). (B) Intersection analysis between the differential gene expression analysis and MITOCARTA3.0 gene set. (C) Correlation analysis between PRELID1 and VDACs. (D) Expression of PRELID1 and VDAC family genes (VDAC1, VDAC2, VDAC3) is shown across spatial clusters across three CRC tissue sections, and co‐expression of all four genes is indicated for spatial points within the tissue. Color intensity represents the degree of co‐localization, with deeper red indicating higher co‐expression. The heatmap shows a continuous scale from 0 to 1, reflecting the relative expression levels of all four genes in each spatial spot. (E‐H) Flow cytometry was used to evaluate Bgc cell responses under different treatment conditions: negative control (NC)‐shPRELID1 (E), NC‐shVDAC3 (F), NC‐sh1PRELID1‐sh1PRELID1 + VDAC3 (G), and NC‐PRELID1‐PRELID1 + sh1VDAC3 (H). *n* = 3 biologically independent samples per group, and statistical significance was determined by unpaired two‐tailed Student's *t*‐test or one‐way ANOVA with post hoc tests. (I) Immunohistochemical analysis was performed to assess the expression of PRELID1 in CD20^+^ cells within colorectal cancer tissues from pCR and non‐pCR patients. Scale bars for 20× figures are 100 µm, and those for 40× figures are 50 µm. Quantification was performed across *n* = 20 patient samples, with statistical significance assessed by unpaired two‐tailed *t*‐test. (J) Representative mIF images showing PRELID1 (red) in CD20^+^ cells (cyan), CD95 (green), and IgD (orange) within CRC tissues from pCR and non‐pCR patients. Scale bars, 50 µm. Quantification of the merged area is shown on the right (*n* = 9 per group, with statistical significance assessed by unpaired two‐tailed *t*‐test). Data are presented as mean ± SEM; ^*^ *p*<0.05, ^**^ *p*<0.01, ^***^ *p*<0.001, ns not significant.

Differential gene expression analysis of Bgc cells from the overall dataset identified the top ten downregulated genes in the pCR group, ranked by log fold change (logFC) (Figure 3A; Figure S7). Further intersection with the MITOCARTA3.0 gene set revealed that among the top ten downregulated proteins, PRELID1, BAX, and VDAC3 are mitochondria‐associated genes (Figure 3B). Flow cytometry analysis demonstrated that knockdown of PRELID1 enhanced Bgc cell responses, as indicated by an increased frequency of IgD^lo^‐ activated B cells and GL7^+^ CD95^+^ B cells, consistent with the Bgc cell phenotype [14]. This effect was reversed by VDAC3 overexpression. Moreover, knockdown of VDAC3 also promoted Bgc cell activation, whereas BAX knockdown did not produce a similar effect (Figure 3E–H; Figure S8). These findings led us to conclude that PRELID1 and VDAC3 cooperatively regulate Bgc cell activation. Accordingly, we focused our subsequent investigations on these two molecules. Meanwhile, correlation analysis revealed an association between PRELID1 and VDAC family genes (VDAC1, VDAC2, VDAC3). Spatial transcriptomics analysis of three colorectal cancer tissue sections (GSM7089856) identified six major spatial clusters, with PRELID1 and VDACs exhibiting broadly high expression across most clusters, and co‐expression of all four genes observed in multiple spatial locations (Figure 3C,D; Figure S9). Notably, this spatial co‐expression indicates regional overlap at the transcript level rather than direct molecular interaction or functional coupling and therefore should be interpreted as suggestive spatial association rather than definitive functional evidence. Moreover, immunohistochemistry (IHC) and mIF jointly confirmed the downregulation of PRELID1 expression in Bgc cells within the pCR group (Figure 3I,J).

### 2.3 Loss of PRELID1 Impairs PINK1/Parkin‐Dependent Mitophagy in Bgc Cells, which can be Partially Rescued by VDAC3 Overexpression

**FIGURE 4:**
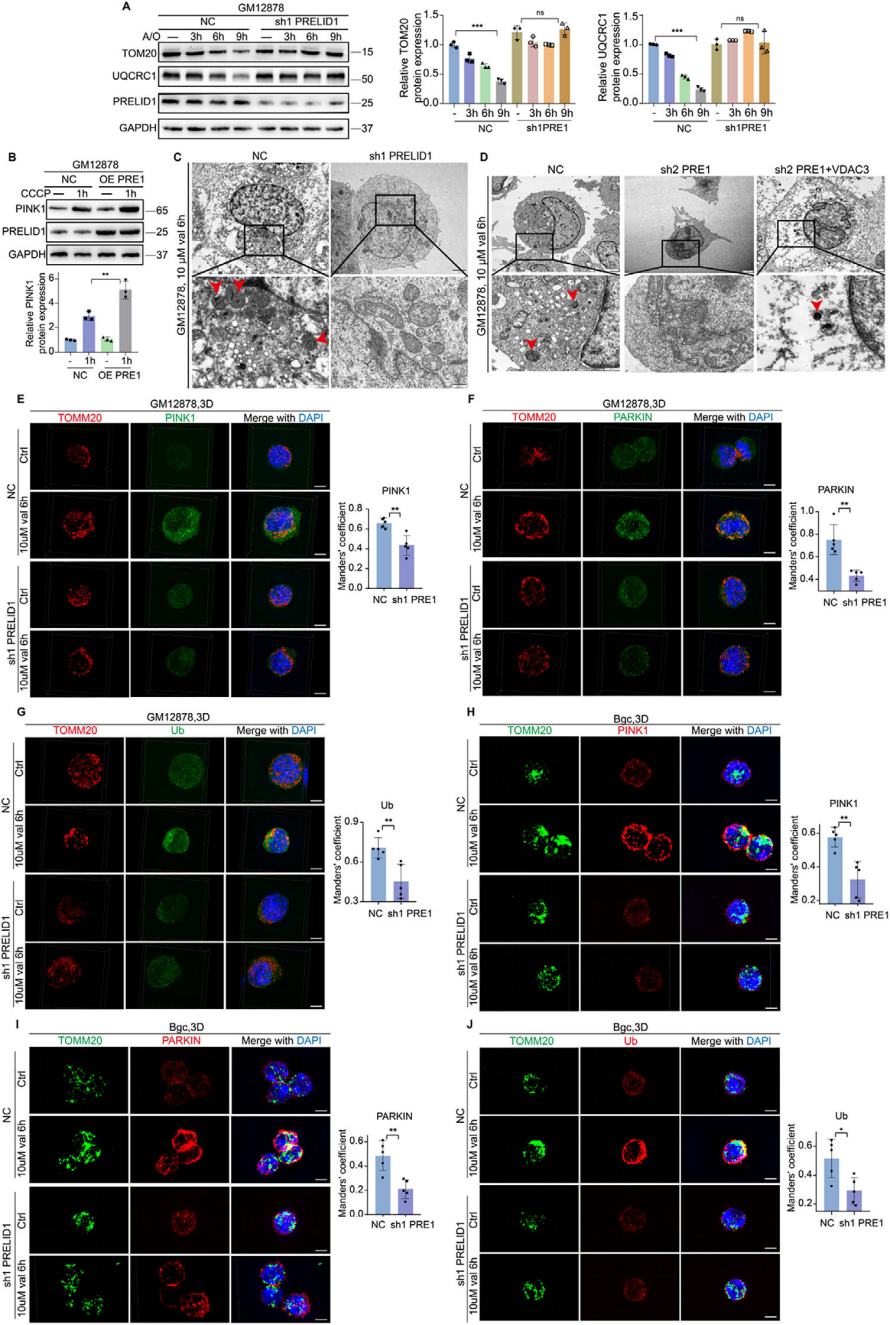
Loss of PRELID1 impairs PINK1/Parkin‐dependent mitophagy in Bgc cells, which can be partially rescued by VDAC3 overexpression. (A) Representative Western blot showing the expression of TOMM20, UQCRC1, and PRELID1 in NC or PRELID1‐knockdown GM12878 cells treated with antimycin A and oligomycin (A/O) for 3, 6, or 9 h. Experiments were performed in *n* = 3 independent replicates. Statistical significance was determined using two‐way repeated‐measures ANOVA. (B) Representative Western blot showing PINK1 expression in NC or PRELID1‐overexpression GM12878 cells treated with or without 7.5 µm CCCP for 1 h. Experiments were performed in *n* = 3 independent replicates. Statistical significance was determined using two‐way repeated‐measures ANOVA. (C,D) Representative transmission electron microscopy (TEM) images showing mitophagy structures in NC Bgc cells, PRELID1‐knockdown Bgc cells, and Bgc cells with PRELID1 knockdown plus VDAC3 overexpression. Scale bars, 500 nm. Experiments were performed in *n* = 3 independent replicates. Statistical significance was determined using one‐way ANOVA. (E–J) Representative immunofluorescence (IF) images of PINK1, PARKIN, TOMM20, and Ub in NC or PRELID1‐knockdown GM12878 and mouse Bgc cells, with or without treatment with 10 µM valinomycin for 6 h acquired using 3D structured illumination microscopy (3D‐SIM). Nuclei were counterstained with DAPI. Experiments were performed in *n* = 5 independent replicates, and fluorescence intensity was quantified using two‐way repeated‐measures ANOVA. Scale bars, 10 µm. Data are presented as mean ± SEM; ^*^ *p*<0.05, ^**^ *p*<0.01, ^***^ *p*<0.001, ns not significant.

**FIGURE 5:**
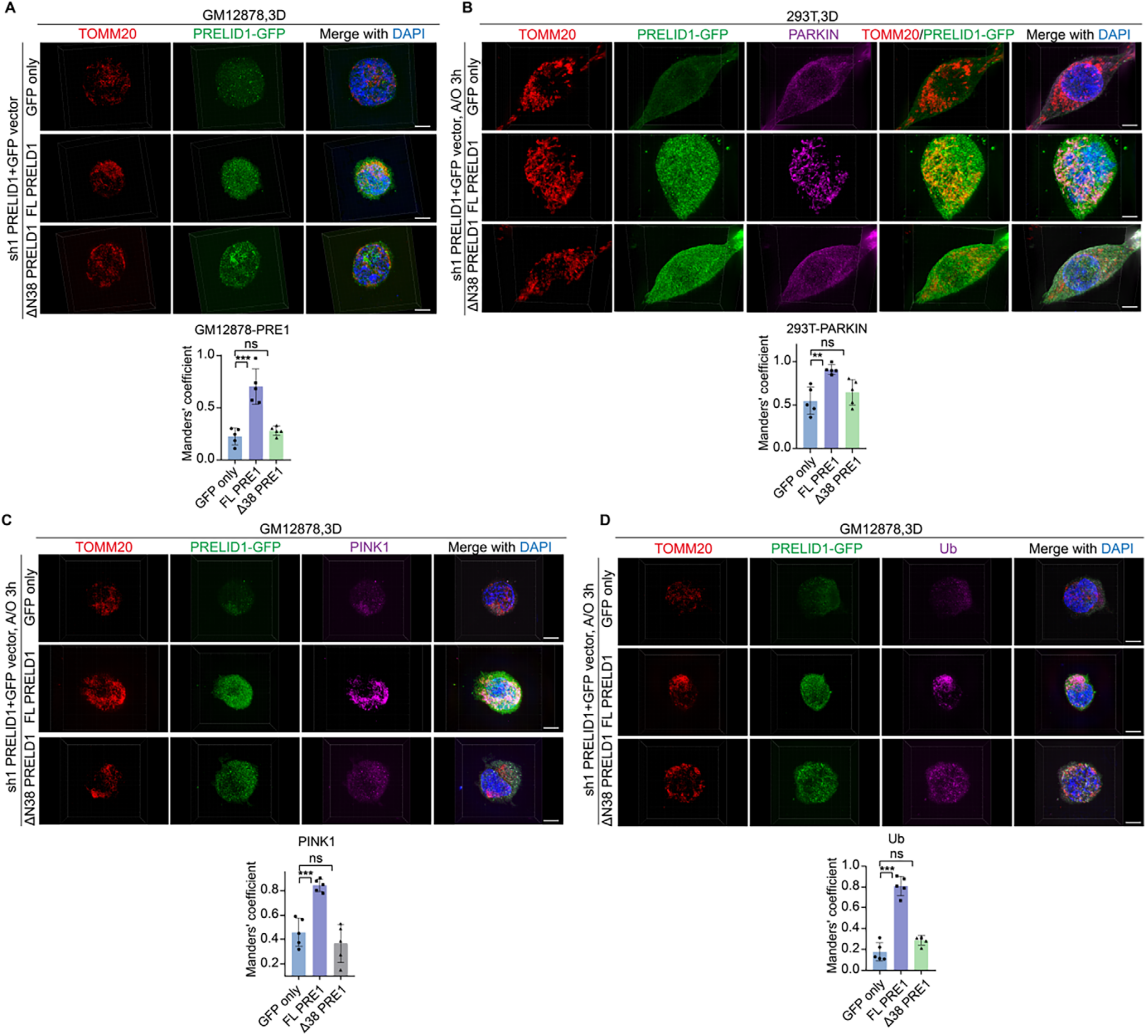
The mitochondrial localization of PRELID1 is essential for PINK1 accumulation and Parkin recruitment. (A–D) Representative images of PRELID1‐GFP, PARKIN, PINK1, Ub, and TOM20 in shPRELID1‐transfected GM12878 or 293T cells overexpressing GFP, FL PRELID1‐GFP, or ∆N38 PRELID1‐GFP, following treatment with antimycin A and oligomycin (A/O) for 3 h, acquired using 3D‐SIM. Nuclei were counterstained with DAPI. Experiments were performed in *n* = 5 independent replicates, and fluorescence intensity was quantified using one‐way ANOVA. Scale bars, 10 µm. Data are presented as mean ± SEM; ^*^ *p*<0.05, ^**^ *p*<0.01, ^***^ *p*<0.001, ns not significant.

Since PRELID1 is a mitochondrial lipid transporter implicated in maintaining mitochondrial integrity, and it cooperates with VDAC3 to regulate Bgc cell activation, we hypothesized that its immunoregulatory role might be linked to mitochondrial homeostasis. Given its mitochondrial localization and potential role in organelle quality control, we specifically focused on PRELID1 to examine whether it influences mitophagy in Bgc cells—a critical process for eliminating damaged mitochondria and sustaining cellular fitness. To this end, we first examined whether PRELID1 affects the initiation of the canonical PINK1/Parkin‐dependent mitophagy pathway. WB analysis showed that A/O treatment induced a time‐dependent reduction of TOMM20 and the mitochondrial matrix protein UQCRC1 in control Bgc cells, which was entirely blocked in PRELID1‐knockdown cells (Figure 4A). WB analysis also revealed that overexpression of PRELID1 accelerated the accumulation of PINK1 during mitochondrial depolarization (Figure 4B). Electron microscopy further confirmed that PRELID1 knockdown reduced mitophagy, whereas VDAC3 overexpression restored this effect (Figure 4C,D). Compared to control GM12878 or Bgc cells, PRELID1 knockdown markedly impaired the mitochondrial recruitment of PINK1, Parkin, and phosphorylated ubiquitin, which are critical steps during PINK1/Parkin‐dependent mitophagy (Figure 4E–J). We next aimed to identify the specific role of PRELID1 involved in facilitating PINK1 accumulation and Parkin recruitment. We overexpressed constructs containing a shPRELID1 sequence and one of the following: C‐terminal GFP‐tagged PRELID1: full‐length PRELID1 (FL PRELID1) and an N‐terminal 38‐amino‐acid deletion mutant [∆N38 PRELID1, which fails to localize on mitochondria due to lack of its N‐terminal hydrophobic mitochondrial binding domain (MBD)]. FL PRELID1, which partially localizes to both mitochondria and cytoplasm consistent with its known subcellular distribution pattern [23], restored the mitochondrial recruitment of PINK1, Parkin, and phospho‐Ub in PRELID1‐knockdown cells, whereas ∆N38 PRELID1, lacking its N‐terminal mitochondrial binding domain, was restricted to the cytoplasm and failed to do so (Figure 5A–D). These findings indicate that PRELID1 functions as a scaffold within mitochondria, a role that is essential for the accumulation of PINK1 and the subsequent recruitment of Parkin.

### 2.4 PRELID1 and VDACs Regulate Mitochondria–Lysosome Interactions via TRPML1 in Bgc Cells, a Process Essential for Lysosomal Repair

**FIGURE 6:**
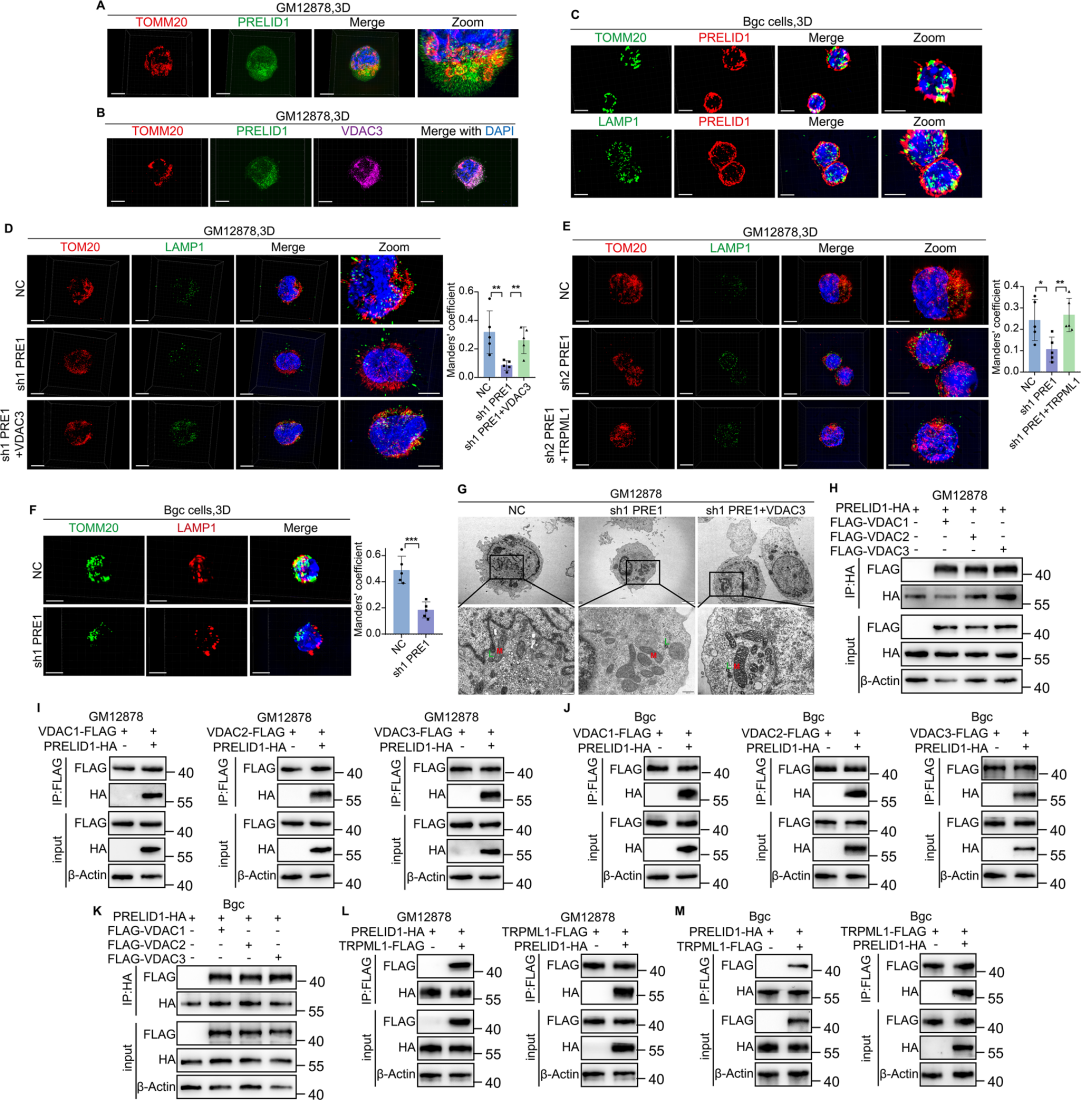
PRELID1 and VDACs regulate mitochondria‐lysosome interactions via TRPML1. (A) Representative images acquired by 3D‐SIM showing the colocalization of TOMM20 and PRELID1 in GM12878 cells. Nuclei were counterstained with DAPI. Scale bar, 10 µm. (B) Representative images acquired by 3D‐SIM showing the colocalization of TOMM20, PRELID1 and VDAC3 in GM12878 cells. Nuclei were counterstained with DAPI. Scale bar, 10 µm. (C) Representative images acquired by 3D‐SIM showing the colocalization of TOMM20/LAMP1 and PRELID1 in mouse Bgc cells. Nuclei were counterstained with DAPI. Scale bar, 10 µm. (D,E) Representative 3D‐SIM images showing mitochondrial‐lysosomal contacts in GM12878 cells. Mitochondria and lysosomes were visualized using TOMM20 and LAMP1 staining, respectively, in NC, PRELID1‐knockdown, PRELID1‐knockdown + VDAC3‐overexpressing, and PRELID1‐knockdown + TRPML1‐overexpressing cells. Nuclei were counterstained with DAPI. Scale bar, 10 µm (*n* = 5 per group, with statistical significance assessed by one‐way ANOVA). (F) Representative 3D‐SIM images showing mitochondrial‐lysosomal contacts in mouse Bgc cells. Mitochondria and lysosomes were labeled with TOMM20 and LAMP1 antibodies, respectively, in the NC and PRELID1‐knockdown groups. Nuclei were counterstained with DAPI. Scale bar, 10 µm. Data are presented as mean ± SEM (*n* = 5 per group, with statistical significance assessed by unpaired two‐tailed *t*‐test). (G) Representative transmission electron microscopy (TEM) images of mitochondrial–lysosomal contacts in GM12878 cells. Images show ultrastructural features in NC, PRELID1‐knockdown, and PRELID1‐knockdown with VDAC3‐overexpression groups. Scale bar, 500 nm. (H‐K) Co‐immunoprecipitation (coIP) analysis of the interaction between PRELID1 and VDACs in GM12878 and mouse Bgc cells. (L,M) CoIP analysis of the interaction between PRELID1 and TRPML1 in GM12878 and mouse Bgc cells. Data are presented as mean ± SEM; ^*^ *p*<0.05, ^**^ *p*<0.01, ^***^ *p*<0.001, ns not significant. [Correction added on 12th February 2026, after first online publication: Figure 6E is updated.]

Given the cooperative role of PRELID1 and VDAC3 in Bgc cell activation, and the observed impairment of mitophagy upon PRELID1 loss, we next explored their role in mitochondrial communication with other organelles. As VDACs and TRPML1 have been implicated in mitochondria‐lysosome interactions [18], we examined whether PRELID1 and VDACs, through their mutual interaction and cooperation with TRPML1, mediate this process in Bgc cells—a potential link between mitochondrial dynamics and immune function. Using 3D structured illumination microscopy (3D‐SIM), we observed colocalization among TOMM20, LAMP1, PRELID1, and VDAC3, providing subcellular spatial evidence that supports the involvement of PRELID1 and VDACs in regulating mitochondria‐lysosome interactions (Figure 6A–C). Using 3D‐SIM, we found that mitochondria‐lysosome contacts were markedly reduced in PRELID1‐knockdown cells compared with negative control (NC) cells, whereas overexpression of either VDAC3 or TRPML1 was able to restore these contacts (Figure 6D–F). Electron microscopy further confirmed this finding (Figure 6G). Co‐immunoprecipitation (coIP) assays further confirmed the interaction between PRELID1 and VDACs (Figure 6H–K). Further coIP experiments revealed an interaction between PRELID1 and TRPML1 (Figure 6L–M). These findings suggest that the interplay among PRELID1, VDACs, and TRPML1 collectively regulates mitochondria‐lysosome interactions.

**FIGURE 7:**
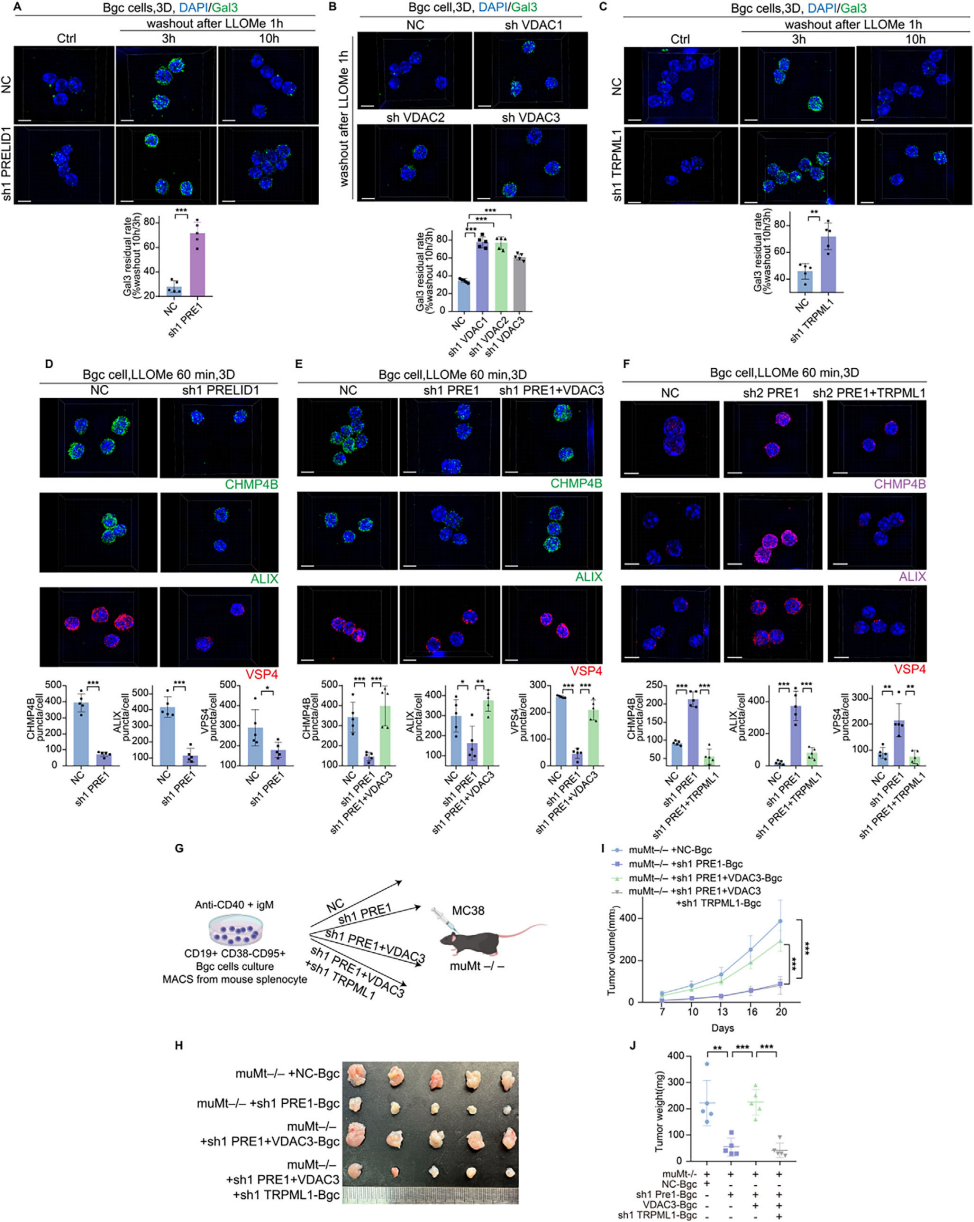
PRELID1 and VDACs regulate mitochondria–lysosome interactions via TRPML1, a process essential for lysosomal repair. (A) Representative immunofluorescence images acquired using 3D‐SIM showing Galectin‐3 (Gal3) puncta in primary mouse Bgc cells with or without PRELID1 knockdown, 10 h after withdrawal of L‐leucyl‐L‐leucine methyl ester (LLOMe) (*n* = 5 per group, with statistical significance assessed by two‐way repeated‐measures ANOVA). (B,C) Representative immunofluorescence images acquired using 3D‐SIM showing Gal3 puncta in primary mouse Bgc cells with knockdown of VDACs (B) or TRPML1 (C), 10 h after withdrawal of LLOMe (*n* = 5 per group, with statistical significance assessed by one‐way ANOVA or two‐way repeated‐measures ANOVA). (D) Representative images acquired by 3D‐SIM showing the recruitment of key ESCRT components to damaged lysosomes in primary mouse Bgc cells. CHMP4B, ALIX, and VPS4 puncta in NC or PRELID1‐knockdown cells (*n* = 5 per group, with statistical significance assessed by unpaired two‐tailed Student's *t*‐test). (E,F) Effects of VDAC3 (E) or TRPML1 (F) overexpression on the recruitment of these components following PRELID1 knockdown (*n* = 5 per group, with statistical significance assessed by one‐way ANOVA). (G) Schematic diagram of experimental design. (H) Representative macroscopic images of tumors from the following groups: muMt^−/−^ + NC‐Bgc, muMt^−/−^ + sh1 PRE1‐Bgc, muMt^−/−^ + sh1 PRE1+VDAC3‐Bgc, muMt^−/−^ + sh1 PRE1+VDAC3+sh1 TRPML1‐Bgc. (I) Tumor growth curves measured over time for each group (*n* = 5 per group, with statistical significance assessed by two‐way repeated‐measures ANOVA). (J) Tumor weights measured on the day of sacrifice for the indicated groups (*n* = 5 per group, with statistical significance assessed by one‐way ANOVA). Data are presented as mean ± SEM; ^*^ *p*<0.05, ^**^ *p*< 0.01, ^***^ *p*<0.001, ns not significant.

As mitochondria‐lysosome interactions are critical for lysosomal repair [15, 17, 27], we next investigated whether the PRELID1‐VDAC‐TRPML1 axis is required for restoring lysosomal function following damage. We knocked down PRELID1 and examined the turnover of damaged lysosomes following treatment with L‐leucyl‐L‐leucine methyl ester (LLOMe), using galectin‐3 (Gal3) as a marker. Gal3, a β‐galactoside‐binding lectin, binds to exposed luminal glycans upon lysosomal membrane damage, leading to the formation of cytosolic puncta [28]. Notably, PRELID1 knockdown impaired the clearance of Gal3 puncta within 10 h after LLOMe withdrawal, indicating that PRELID1 is essential for the removal of damaged lysosomes (Figure 7A). Similarly, knockdown of VDACs and TRPML1 also impaired the clearance of damaged lysosomes (Figure 7B,C). Subsequently, we investigated the potential role of PRELID1 in the repair process mediated by the endosomal sorting complexes required for transport (ESCRT), which plays a pivotal role in restoring lysosomal homeostasis following injury [29, 30]. Notably, depletion of PRELID1 hindered the recruitment of key ESCRT components to damaged lysosomes, as indicated by decreased puncta of CHMP4B (an ESCRT‐III subunit), ALIX (an interactor of CHMP4A), and VPS4 (an ESCRT‐III‐associated protein). This effect was reversed by the overexpression of VDAC3 or TRPML1 (Figure 7D–F). To investigate the potential connection between mitochondria–lysosome interactions and immune function, we performed a series of in vivo experiments. Subcutaneous tumor formation experiments demonstrated that overexpression of VDAC3 reversed the tumor growth suppression induced by the adoptive transfer of PRELID1‐knockdown Bgc cells, whereas subsequent knockdown of TRPML1 again led to a reduction in tumor growth (Figure 7G–J). Flow cytometry of tumor‐infiltrating lymphocytes from subcutaneous tumors consistently showed altered expression of exhaustion and cytotoxicity markers in CD8^+^ T cells (Figure S10).

### 2.5 By Regulating Mitochondria‐Lysosome Interactions, PRELID1 and VDACs Drive a Senescence‐Like State in Bgc Cells

**FIGURE 8:**
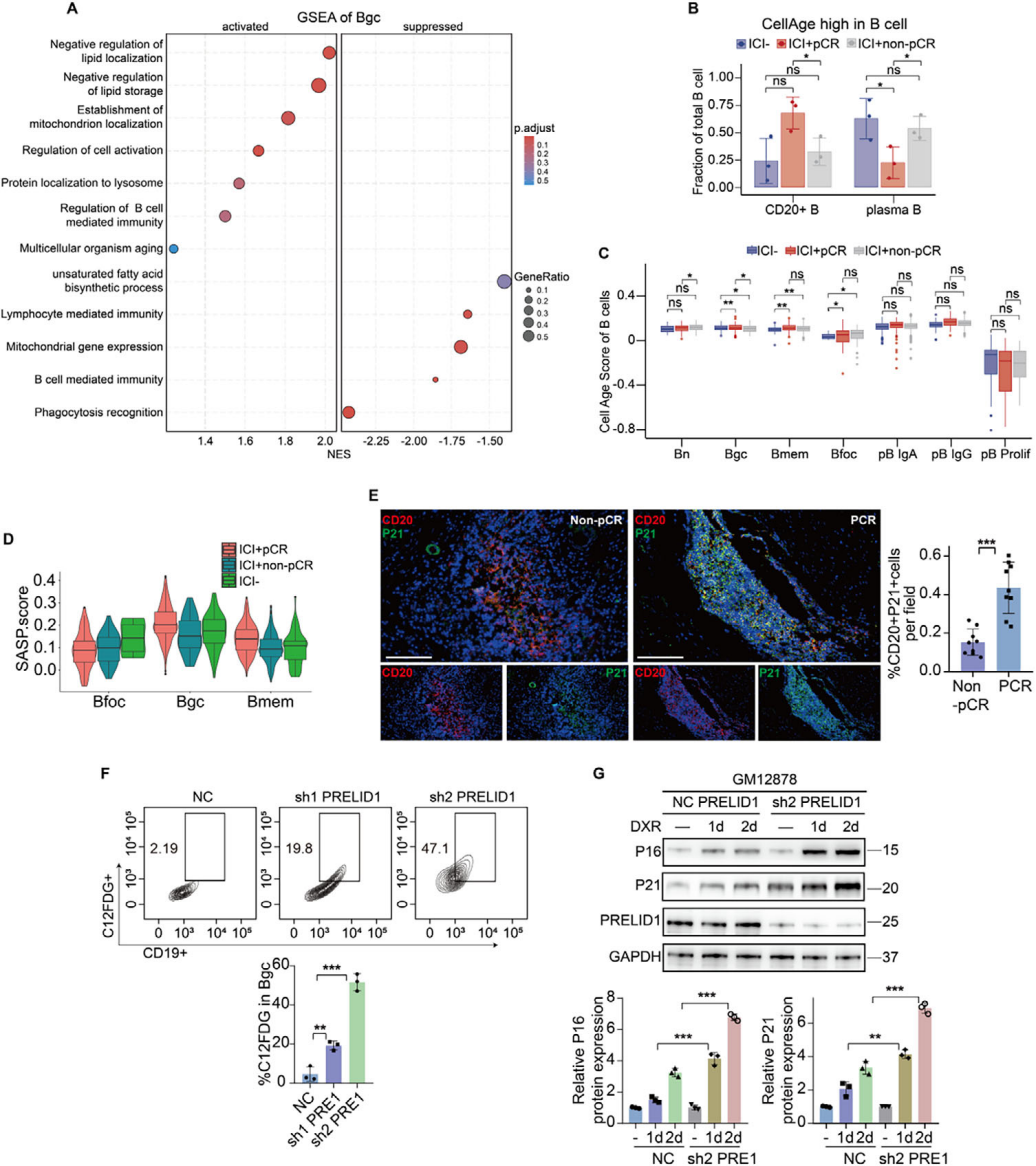
By regulating mitochondria‐lysosome interactions, PRELID1 and VDACs drive a senescence‐like state in Bgc cells. (A) GSEA pathway enrichment analysis of Bgc cells selected from the overall dataset. (B) Analysis of the proportion of CellAge‐high cells among B cells across the ICI^−^, ICI^+^pCR, and ICI^+^non‐pCR groups. (C) Cell Age Score of distinct B cell clusters across the ICI–, ICI^+^pCR, and ICI^+^non‐pCR groups. (D) secretory phenotype (SASP) Score of distinct B cell clusters across the ICI^−^, ICI^+^pCR, and ICI^+^non‐pCR groups. (E) Representative multiplex immunofluorescence images showing P21 expression on CD20^+^ cells. Quantification of the merged area between CD20^+^ and P21 signals is shown on the right. Scale bars, 50 µm (*n* = 9 per group, with statistical significance assessed by unpaired two‐tailed Student's *t*‐test). (F) Flow cytometry combined with C12FDG staining (a β‐galactosidase probe) was performed to assess the regulatory effect of PRELID1 knockdown on the senescence level of Bgc cells (*n* = 3 per group, with statistical significance assessed by one‐way ANOVA). (G) WB analysis was performed to examine the regulatory effect of PRELID1 knockdown on the senescence level of Bgc cells, focusing on the expression changes of senescence‐associated markers P16 and P21 to evaluate their role in the regulation of cellular senescence (*n* = 3 per group, with statistical significance assessed by two‐way repeated‐measures ANOVA). Data are presented as mean ± SEM; ^*^ *p*<0.05, ^**^ *p*<0.01, ^***^ *p*<0.001, ns not significant.

Building on our findings that PRELID1 and VDACs regulate mitochondria‐lysosome interactions in Bgc cells, we next explored the functional consequences of this regulation. Given that mitochondrial and lysosomal dysfunction is tightly linked to cellular senescence, we hypothesized that disruption of this axis may contribute to a senescence‐like phenotype in Bgc cells. GSEA pathway enrichment analysis of Bgc cells selected from the overall dataset revealed significant enrichment of pathways related to mitochondria, lysosomes, and cellular senescence in association with anti‐PD‐1 efficacy (Figure 8A). Senescence‐related analysis revealed that Bgc cells in the pCR group exhibited a higher level of senescence compared to those in the non‐pCR group (Figure 8B–D). Immunohistochemistry and multiplex immunofluorescence analysis further validated this finding (Figure 8E; Figure S11A). Furthermore, we assessed senescence levels of Bgc cells by C12FDG staining (a β‐galactosidase probe). Flow cytometry analysis showed that knockdown of BAX did not alter the senescence level of Bgc cells, whereas knockdown of PRELID1 or VDAC3 significantly affected Bgc cell senescence (Figure 8F; Figure S11B,C). To further validate these findings at the protein level, we performed WB analysis to examine the expression of classical senescence markers. Consistent with the flow cytometry results, PRELID1 knockdown led to a marked upregulation of P16 and P21 in Bgc cells, supporting the notion that PRELID1 is critically involved in the regulation of Bgc cell senescence (Figure 8G). Based on previous reports (Potting et al., Cell Metab., 2013) [31], loss of PRELID1 typically disrupts mitochondrial phospholipid transport and induces apoptosis rather than senescence. To investigate the effect of PRELID1 knockdown on Bgc cells, apoptosis markers including P53, cleaved caspase‐3, and cytochrome c were assessed by Western blot, and Annexin V/PI staining was performed (Figure S12). No notable changes in apoptosis marker expression or Annexin V/PI staining were observed in PRELID1 knockdown cells compared with control, indicating that PRELID1 depletion induces a senescence‐like cellular state rather than apoptosis in Bgc cells.

### 2.6 The Senescence‐Like State in Bgc Cells Driven by PRELID1 and VDACs Alleviates CD8^+^ T Cell Exhaustion

**FIGURE 9:**
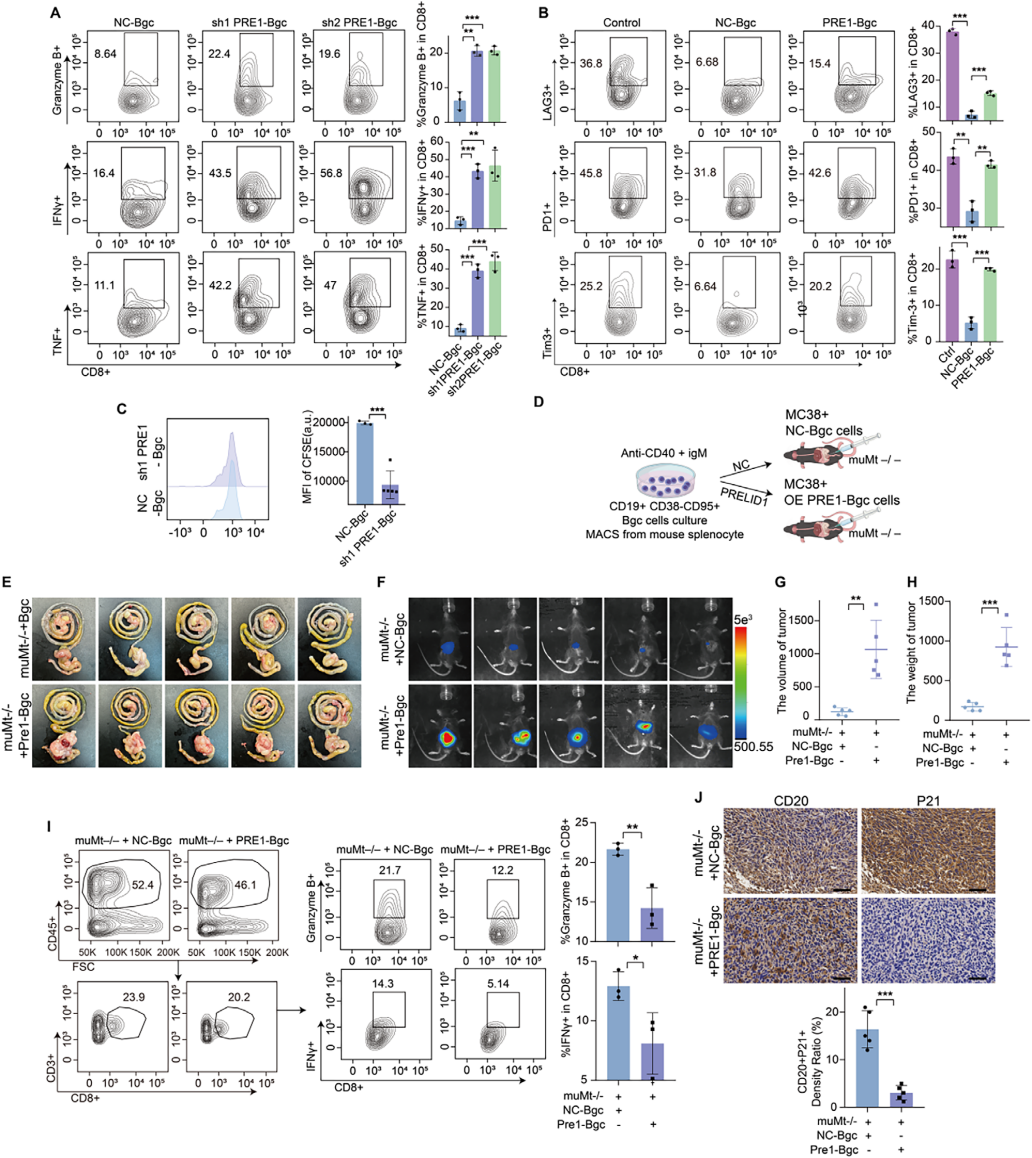
PRELID1 suppresses Bgc cell senescence and impairs CD8^+^ T cell function, thereby promoting tumor progression. (A) Flow cytometry analysis of cytotoxicity markers (Granzyme B, IFNγ and TNF) on CD8^+^ T cells (*n* = 3 per group, with statistical significance assessed by one‐way ANOVA). (B) Flow cytometry analysis of exhaustion markers (PD‐1, LAG3, and Tim‐3) on CD8^+^ T cells (*n* = 3 per group, with statistical significance assessed by one‐way ANOVA). (C) CFSE dilution assay showing proliferation of CD8^+^ T cells in co‐culture with Bgc cells. (D) Schematic illustration of the adoptive transfer of Bgc cells into muMt^−/−^ mice in an in situ colorectal cancer model. (E) Representative gross images of cecal tumors from muMt^−/−^ mice with NC or PRELID1‐overexpressing Bgc cell transfer. (F) Representative bioluminescence imaging showing tumor burden in each group on the day of sacrifice. (G) Tumor volumes measured on the day of sacrifice (*n* = 5 per group, with statistical significance assessed by unpaired two‐tailed Student's *t*‐test). (H) Tumor weights measured on the day of sacrifice (*n* = 5 per group, with statistical significance assessed by unpaired two‐tailed Student's *t*‐test). (I) Flow cytometry analysis of tumor‐infiltrating lymphocytes showing expression levels of effector function markers (Granzyme B, IFNγ) on CD8^+^ T cells (*n* = 3 per group, with statistical significance assessed by unpaired two‐tailed Student's *t*‐test). (J) Immunohistochemical analysis of P21 expression in CD20^+^ B cells from cecal orthotopic tumors of mice receiving adoptive transfer of NC‐Bgc or PRELID1‐overexpressing Bgc cells. Tumor sections were stained for CD20 and P21, and representative images are shown (*n* = 5 per group, with statistical significance assessed by unpaired two‐tailed Student's *t*‐test). Scale bars for 40× figures are 50 µm. Data are presented as mean ± SEM; ^*^ *p*<0.05, ^**^ *p*<0.01, ^***^ *p*<0.001, ns not significant.

**FIGURE 10:**
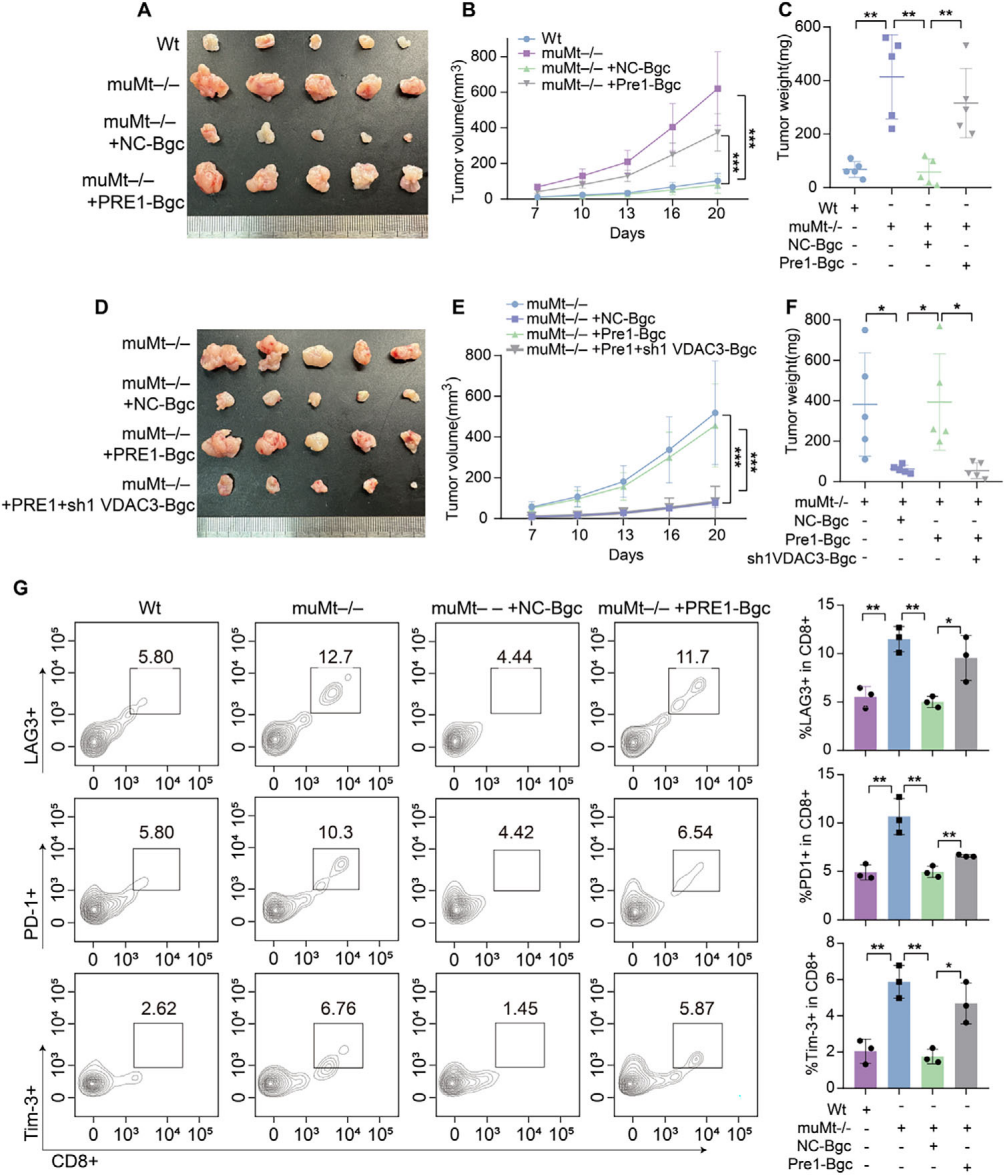
PRELID1‐overexpressing Bgc cells exacerbate tumor progression and CD8^+^ T cell exhaustion, which is mitigated by VDAC3 knockdown. (A) Representative images of subcutaneous tumors showing treatment groups: Wt, muMt^−/−^ without‐Bgc, muMt^−/−^ plus NC‐Bgc, and muMt^−/−^ plus PRELID1‐overexpressing Bgc. (B) Tumor volume growth curves over time (*n* = 5 per group, with statistical significance assessed by two‐way repeated‐measures ANOVA). (C) Tumor weights were measured on the day of sacrifice (*n* = 5 per group, with statistical significance assessed by one‐way ANOVA). (D) Representative images of subcutaneous tumors showing treatment groups: muMt^−/−^ without‐Bgc, muMt^−/−^ plus NC‐Bgc, muMt^−/−^ plus PRELID1‐overexpressing Bgc, and muMt^−/−^ plus PRELID1‐overexpressing Bgc with VDAC3 knockdown. (E) Tumor volume growth curves over time (*n* = 5 per group, with statistical significance assessed by two‐way repeated‐measures ANOVA). (F) Tumor weights measured on the day of sacrifice. (*n* = 5 per group, with statistical significance assessed by one‐way ANOVA). (G) Flow cytometry analysis of tumor‐infiltrating lymphocytes showing expression levels of exhaustion markers (PD‐1, LAG3, TIM‐3) on CD8^+^ T cells (*n* = 3 per group, with statistical significance assessed by one‐way ANOVA). Data are presented as mean ± SEM; ^*^ *p*<0.05, ^**^ *p*<0.01, ^***^ *p*<0.001, ns not significant.

Since Bgc cells engage in immunoregulatory crosstalk with CD8^+^ T cells, we investigated whether the senescence‐like state driven by PRELID1 and VDACs affects CD8^+^ T cell exhaustion. Co‐culture experiments demonstrated that PRELID1‐knockdown Bgc cells enhanced the effector function of CD8^+^ T cells (Figure 9A; Figure S13A), reduced their exhaustion phenotype (Figure 9B; Figure S13B), and promoted their proliferation (Figure 9C). This effect was reversed by VDAC3 overexpression (Figure S13C,D). In an in situ tumor model, adoptive transfer of Bgc cells overexpressing PRELID1 significantly accelerated tumor growth (Figure 9D–H). Analysis of tumor‐infiltrating lymphocytes by flow cytometry showed that CD8^+^ T cells from mice receiving adoptive transfer of PRELID1‐overexpressing Bgc cells exhibited lower levels of effector function compared to those receiving NC‐Bgc cells (Figure 9I). IHC results showed that CD20^+^ B cells in cecal orthotopic tumors of mice receiving adoptive transfer of PRELID1‐overexpressing Bgc cells exhibited lower levels of P21 expression compared to those receiving NC‐Bgc cells (Figure 9J). To further confirm this finding, we performed a series of in vivo rescue experiments. The subcutaneous tumor model further confirmed that adoptive transfer of PRELID1‐overexpressing Bgc cells significantly accelerated tumor growth, whereas VDAC3 knockdown reversed this effect (Figure 10A–F). Consistently, flow cytometry analysis of tumor‐infiltrating lymphocytes from the subcutaneous tumors revealed a corresponding changes in exhaustion and function marker expression on CD8^+^ T cells (Figure 10G; Figure S6C).

### 2.7 The Senescence‐Like State in Bgc Cells Promotes IL‐7 Secretion, Which Enhances Antitumor Immunity in CRC

**FIGURE 11:**
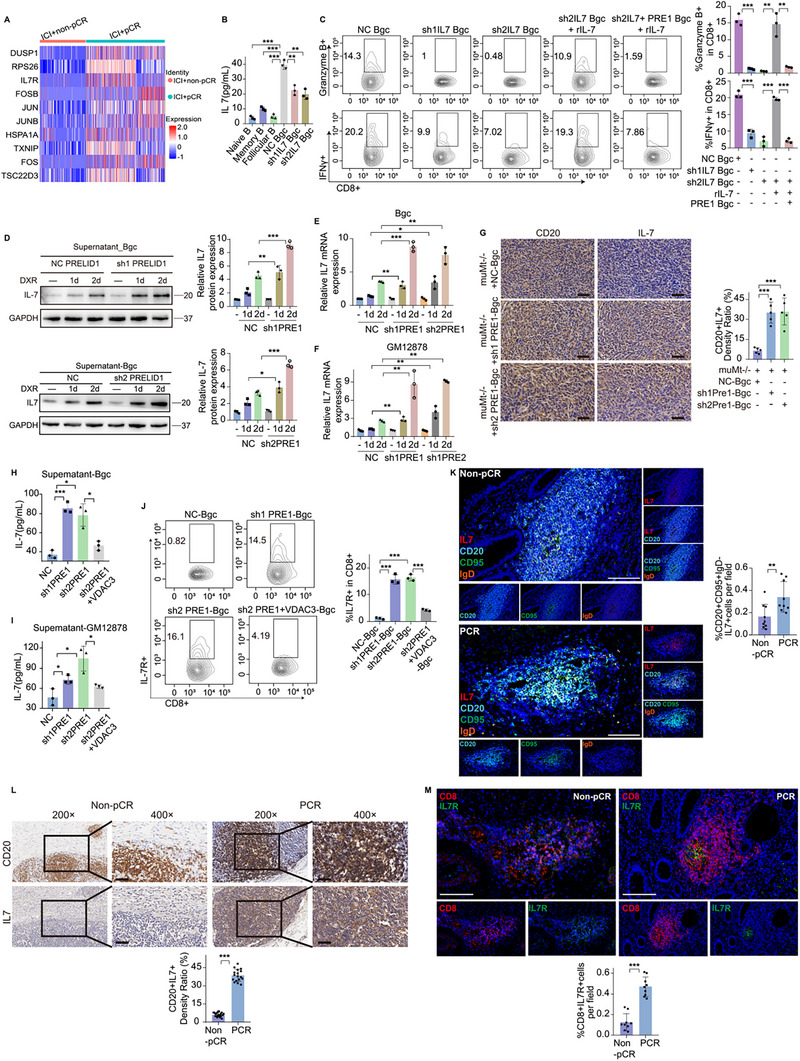
PRELID1 knockdown induces IL‐7 secretion in Bgc cells, and this effect is reversed by VDAC3 overexpression. (A) Differential gene expression analysis was performed to identify the top ten upregulated proteins of CD8^+^ T cells in the pCR group based on log fold change (logFC). (B) ELISA analysis of IL‐7 concentrations in the supernatants of different B‐cell subsets, including naïve B cells, memory B cells, FO B cells, and Bgc cells with or without IL7 knockdown (shIL7‐1 and shIL7‐2). *n* = 3; Statistical significance determined by one‐way ANOVA as appropriate. (C) Flow cytometric assessment of CD8^+^ T‐cell cytotoxicity following co‐culture with Bgc cells under different conditions: with or without IL‐7 knockdown (shIL7‐1 and shIL7‐2), with or without recombinant IL‐7 rescue, and with or without PRELID1 overexpression. *n* = 3. Statistical significance determined by one‐way ANOVA as appropriate. (D) Representative immunoblots showing quantification of IL‐7 concentration in the supernatant of NC or PRELID1‐knockdown (sh1PRE1 and sh2PRE1) primary mouse Bgc cells treated with doxorubicin (DXR) for 3 or 6 days (*n* = 3 per group, with statistical significance assessed by two‐way repeated‐measures ANOVA). (E‐F) Quantitative PCR analysis of IL‐7 mRNA expression in GM12878 cells and murine primary Bgc cells following PRELID1 knockdown (sh1PRE1 and sh2PRE1). Data are shown for control (NC) and PRELID1 knockdown groups. Gene expression levels were normalized to GAPDH (*n* = 3 per group, with statistical significance assessed by two‐way repeated‐measures ANOVA). (G) IHC analysis of IL‐7 expression in intratumoral CD20^+^ B cells from an orthotopic cecal tumor model (*n* = 5 per group, with statistical significance assessed by one‐way ANOVA). (H‐I) ELISA assays measuring IL‐7 levels in the supernatants of primary mouse Bgc cells and GM12878 cells with or without PRELID1 knockdown (sh1PRE1 and sh2PRE1), and with or without VDAC3 overexpression (*n* = 3 per group, with statistical significance assessed by one‐way ANOVA). (J) Flow cytometry analysis of IL‐7R expression in CD8^+^ T cells cocultured with NC Bgc cells, PRELID1‐knockdown (sh1PRE1 and sh2PRE1) Bgc cells, and with or without VDAC3 overexpression Bgc cells (*n* = 3 per group, with statistical significance assessed by one‐way ANOVA). (K) Representative mIF images showing CD20 (cyan), CD95 (green), IgD (orange), and IL‐7 (red) in colorectal cancer tissues from pCR and non‐pCR patients (*n* = 9 per group, with statistical significance assessed by unpaired two‐tailed Student's *t*‐test). Scale bars, 50 µm. (L) Immunohistochemical analysis was performed to assess the expression of IL‐7 in CD20^+^ cells within colorectal cancer tissues from pCR and non‐pCR patients (*n* = 20 per group, with statistical significance assessed by unpaired two‐tailed Student's *t*‐test). Scale bars for 20× figures are 100 µm, and those for 40× figures are 50 µm. (M) Representative mIF images showing the expression of IL‐7R in CD8^+^ cells within CRC tissues from pCR and non‐pCR patients (*n* = 9 per group, with statistical significance assessed by unpaired two‐tailed Student's *t*‐test). Scale bars, 50 µm. Data are presented as mean ± SEM; ^*^ *p*<0.05, ^**^ *p*<0.01, ^***^ *p*<0.001, ns not significant.

**FIGURE 12:**
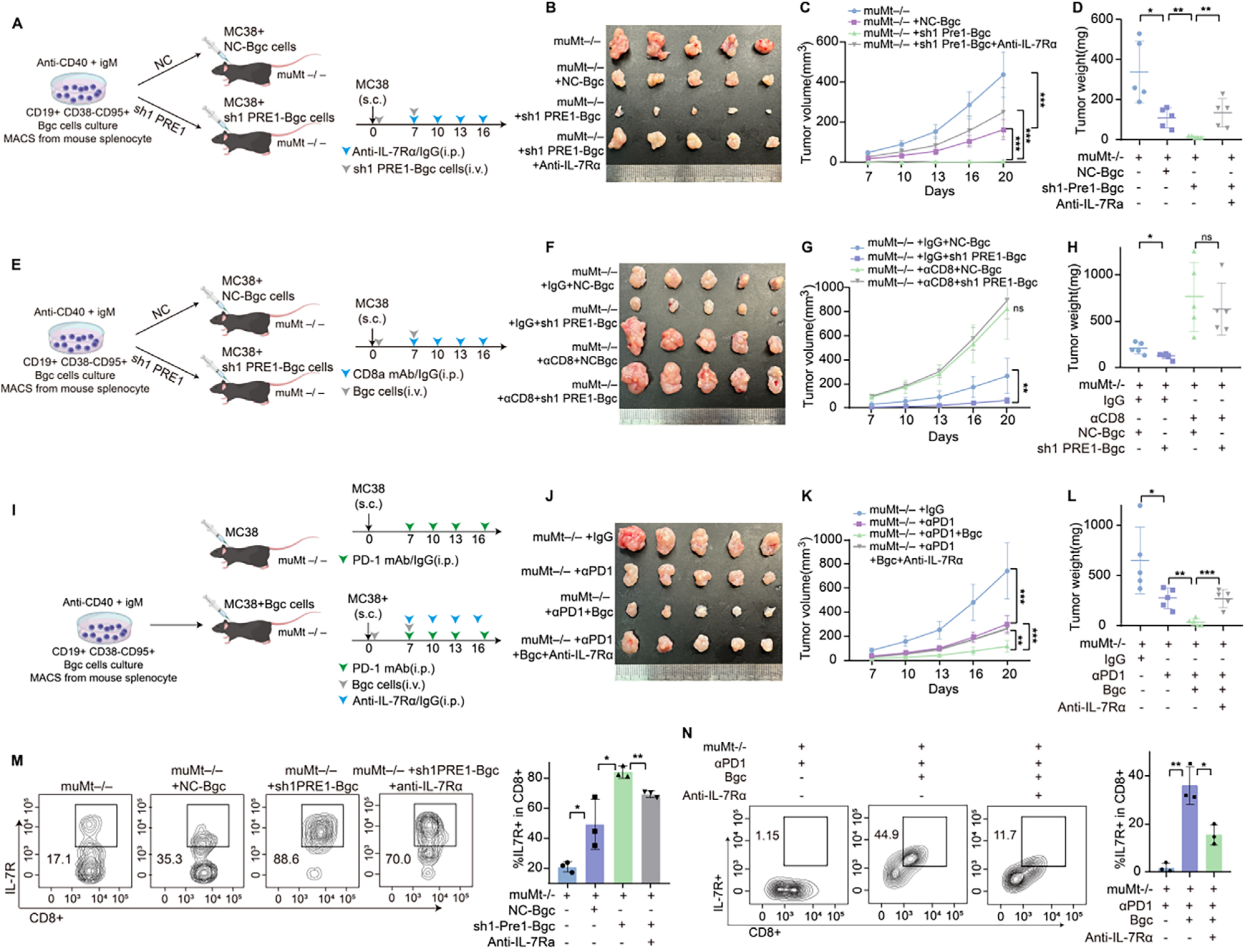
IL‐7/IL‐7R signaling underlies the antitumor function of PRELID1‐deficient senescent‐like Bgc cells and boosts anti‐PD‐1 efficacy via CD8^+^ T cells. (A) Schematic diagram of experimental design. (B) Representative macroscopic images of tumors from the following groups: muMt^−/−^, muMt^−/−^ + NC‐Bgc, muMt^−/−^ + sh1 PRELID1‐Bgc, muMt^−/−^ + sh1 PRELID1‐Bgc + anti‐IL‐7Rα. (C) Tumor growth curves measured over time for each group (*n* = 5 per group, with statistical significance assessed by two‐way repeated‐measures ANOVA). (D) Tumor weights measured on the day of sacrifice for the indicated groups (*n* = 5 per group, with statistical significance assessed by one‐way ANOVA). (E) Schematic diagram of the experimental design. (F) Representative macroscopic images of tumors from the following groups: muMt^−/−^ + IgG + NC‐Bgc, muMt^−/−^ + IgG + sh1 PRELID1‐Bgc, muMt^−/−^ + αCD8 + NC‐Bgc, muMt^−/−^ + αCD8 + sh1 PRELID1‐Bgc. (G) Tumor growth curves measured over time for each group (*n* = 5 per group, with statistical significance assessed by two‐way repeated‐measures ANOVA). (H) Tumor weights measured on the day of sacrifice for the indicated groups (*n* = 5 per group, with statistical significance assessed by one‐way ANOVA). (I) Schematic diagram of experimental design. (J) Representative macroscopic images of tumors from the following groups: muMt^−/−^ + IgG, muMt^−/−^ + αPD1, muMt^−/−^ + αPD1 + Bgc, muMt^−/−^ + αPD1 + Bgc + anti‐IL‐7Rα. (K) Tumor growth curves measured over time for each group (*n* = 5 per group, with statistical significance assessed by two‐way repeated‐measures ANOVA). (L) Tumor weights measured on the day of sacrifice for the indicated groups (*n* = 5 per group, with statistical significance assessed by one‐way ANOVA). (M‐N) Flow cytometry analysis of IL‐7R expression levels in tumor‐infiltrating CD8^+^ T cells from each treatment group (*n* = 3 per group, with statistical significance assessed by one‐way ANOVA). Data are presented as mean ± SEM; ^*^ *p*<0.05, ^**^ *p*<0.01, ^***^ *p*<0.001, ns not significant.

Given that the senescence‐like state in Bgc cells alleviates CD8^+^ T cell exhaustion, we next sought to elucidate the underlying mechanism. CD8^+^ T cells were selected from the overall dataset, and differential gene expression analysis identified the top ten upregulated proteins in the pCR group based on log fold change (logFC). Among these, IL‐7R, a chemokine receptor, was identified as the third most upregulated protein (Figure 11A). To assess the functional relevance of Bgc‐derived IL‐7, IL‐7 secretion levels were measured by ELISA across different B‐cell subsets. Bgc cells exhibited higher IL‐7 production than naïve, memory, or FO B cells, whereas IL7 knockdown (shIL7‐1 and shIL7‐2) significantly reduced IL‐7 levels in the supernatant (Figure 11B). Functionally, the co‐culture of Bgc cells with CD8^+^ T cells revealed decreased cytotoxicity following IL7 knockdown, which was partially restored by recombinant IL‐7 supplementation. However, this rescue effect was attenuated when PRELID1 was overexpressed in IL7‐deficient Bgc cells (Figure 11C). In Bgc cells from the NC group, doxorubicin (DXR) treatment for 3 and 6 days induced cellular senescence, which was accompanied by increased secretion of IL‐7 in the culture supernatant. Knockdown of PRELID1 further augmented this effect, leading to an additional increase in IL‐7 secretion (Figure 11D). QPCR confirmed that knockdown of PRELID1 upregulated IL‐7 mRNA expression in both GM12878 cells and murine primary Bgc cells (Figure 11E,F). Next, using an orthotopic cecal tumor model in mice, we performed adoptive transfer of PRELID1‐knockdown Bgc cells or NC Bgc cells. IHC analysis confirmed that tumors receiving PRELID1‐knockdown Bgc cells exhibited elevated IL‐7 expression specifically in intratumoral B cells (Figure 11G). Further validation using ELISA confirmed that PRELID1 knockdown promoted IL‐7 secretion by mouse primary Bgc cells and GM12878 cells, whereas overexpression of VDAC3 reversed this effect (Figure 11H,I). Flow cytometry analysis accordingly confirmed that coculture with PRELID1‐knockdown Bgc cells upregulated IL‐7R expression in CD8^+^ T cells, whereas this effect was reversed by VDAC3 overexpression (Figure 11J). IHC and mIF jointly confirmed the upregulation of IL‐7 expression in Bgc cells within the pCR group (Figure 11K,L). Correspondingly, mIF confirmed the upregulation of IL‐7R expression in CD8^+^ cells within the pCR group (Figure 11M). Subcutaneous tumor formation experiments demonstrated that administration of anti‐IL‐7Rα antibody reversed the tumor growth suppression induced by the adoptive transfer of PRELID1‐knockdown Bgc cells (Figure 12A–D). To assess whether the tumor‐suppressive effect of PRELID1 knockdown in Bgc cells was dependent on CD8^+^ T cells, we administered anti‐CD8 antibodies in a murine model. CD8^+^ T cell depletion abrogated the inhibitory effect of PRELID1‐deficient Bgc cells on tumor growth (Figure 12E–H). Next, we treated MC38 tumor‐bearing mice with a combination of Bgc cells and anti‐PD‐1 monoclonal antibody. Compared to monotherapy group, the combination therapy resulted in slower MC38 tumor growth. However, this inhibitory effect was reversed by Anti‐IL‐7Rα (Figure 12I–L). Flow cytometry analysis revealed corresponding changes in IL‐7R expression levels in tumor‐infiltrating CD8^+^ T cells (Figure 12M,N).

## 3 Discussion

A portion of current studies on the mechanisms of tumor immunotherapy focus on the influence of tumor cells on the immune microenvironment [32, 33]. Although mechanistic research on tumor immunotherapy has begun to focus more on immune cells [34, 35, 36, 37, 38], the role and mechanisms of B cell subsets in modulating anti‐PD‐1 efficacy remain largely unclear. This study is the first to demonstrate that germinal center B (Bgc) cells in a senescence‐like state can promote antitumor immunity via IL‐7 secretion in CRC.

Although PRELID1, a mitochondrial phospholipid transporter, is known to be essential for mitochondrial membrane integrity and cardiolipin biosynthesis [23, 39, 40], its knockdown in Bgc cells resulted in suppressed mitophagy, yet concurrently promoted Bgc cell responses. This outcome reflected a compensatory immunometabolic adaptation.

While current studies have largely overlooked organelle interactions in B cells within the TME [17, 35, 41, 42], this study reveals that PRELID1 and VDACs regulate mitochondria‐lysosome interactions in Bgc cells. These findings uncover a previously unrecognized organelle‐level regulatory mechanism in B cells, offering new insights into how subcellular dynamics contribute to antitumor immunity. Importantly, this organelle regulatory axis represents the primary mechanistic innovation of the study, linking B cell‐intrinsic metabolic control to immune niche function. Although our focus was on Bgc cells, similar mitochondria–lysosome crosstalk has been reported in T cells [35], where it can influence activation, effector function, and metabolic adaptation. This suggests that organelle‐level regulation may serve as a generalizable immunometabolic checkpoint across immune cell types, shaping functional responses within the TME.

Multiple studies have demonstrated that disruption of mitochondrial‐lysosomal homeostasis can lead to cellular senescence [17]. Furthermore, senescent‐like cells have been reported to exhibit heightened immunostimulatory properties under certain conditions [43, 44]. However, the relationship between B cell senescence and tumor immune activation has not yet been reported. Our study found that the senescence‐like state in Bgc cells driven by PRELID1 and VDACs alleviates CD8^+^ T cell exhaustion and enhances antitumor immunity in CRC. Senescence‐like″ state observed in Bgc cells differs from canonical cellular senescence in that it is not associated with irreversible cell‐cycle arrest or loss of viability, but rather reflects a stress‐adaptive, metabolically reprogrammed state characterized by altered organelle dynamics and enhanced immunostimulatory function, including sustained IL‐7 production. This senescence‐like phenotype supports immune activation within the tumor microenvironment rather than promoting tissue degeneration. Here, the senescence‐like Bgc state serves as the functional outcome of PRELID1–VDAC3–mediated organelle regulation, rather than an independent parallel mechanism.

**FIGURE 13:**
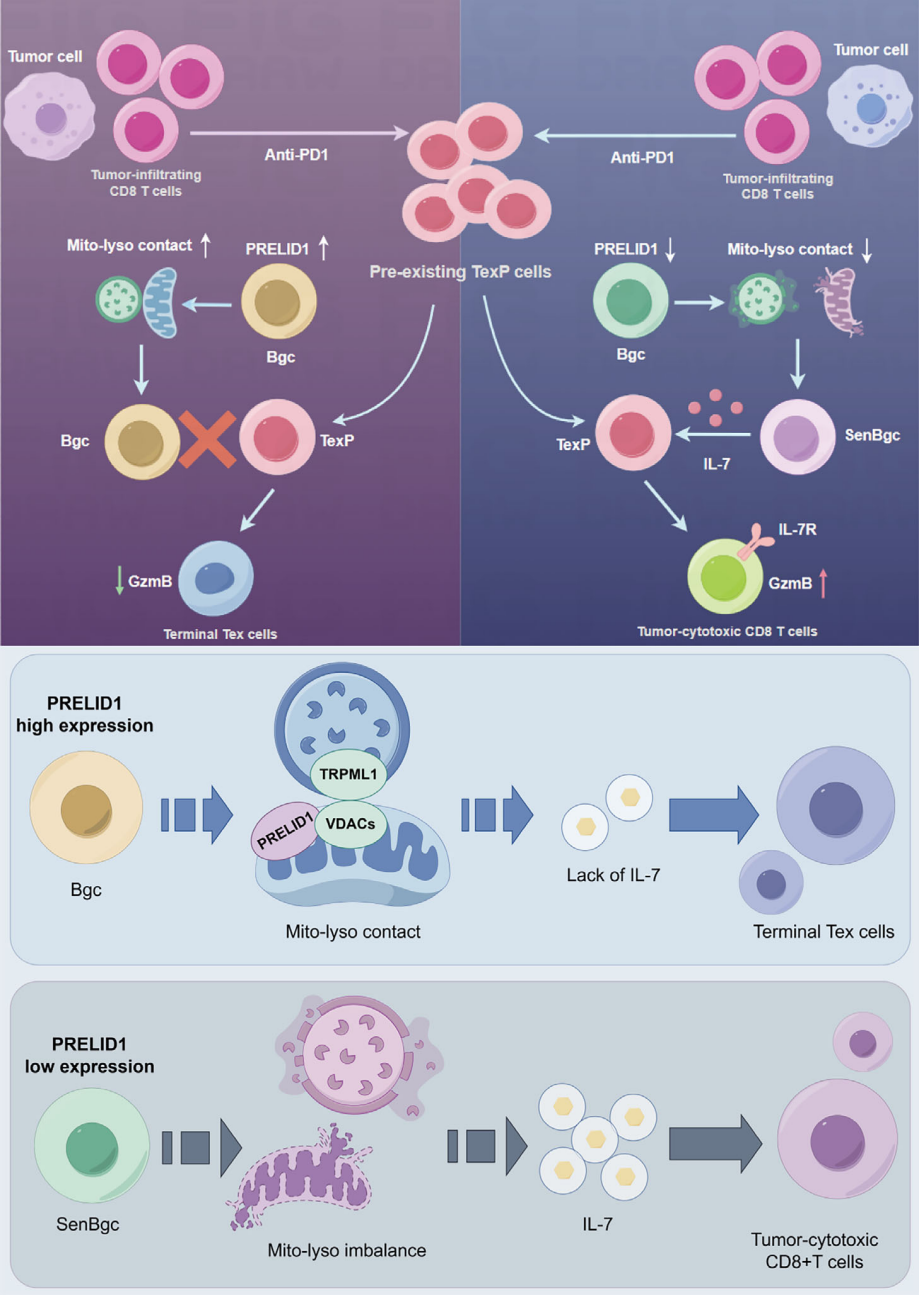
PRELID1 and VDAC3 cooperatively regulate mitochondria‐lysosome interactions to drive senescence‐like Bgc cells that enhance antitumor immunity. Mechanism diagram illustrating how PRELID1 and VDAC3 cooperatively regulate mitochondria‐lysosome interactions via TRPML1 in germinal center B cells, driving a senescence‐like state that enhances IL‐7 secretion and promotes crosstalk with CD8^+^ T cells to sustain antitumor immunity. The upper panel uses distinct background colors to indicate clinical response states: purple represents the non‐pCR condition, and blue represents the pCR condition, illustrating response‐associated differences in Bgc cell‐intrinsic regulation and downstream CD8^+^ T cell modulation.

Overall, we identify a senescence‐like state in Bgc cells that enhances antitumor immunity in CRC by promoting IL‐7 secretion and alleviating CD8^+^ T cell exhaustion. Mechanistically, this senescence‐like state is driven by PRELID1 and VDAC3 through their cooperative regulation of mitochondria‐lysosome interactions in Bgc cells, which enhances IL‐7 secretion and promotes functional crosstalk with CD8^+^ T cells to sustain antitumor immunity (Figure 13). Thus, the conceptual core of this study is the discovery that B cell‐intrinsic organelle regulation establishes a senescence‐like immune‐supportive niche that potentiates IL‐7–dependent CD8^+^ T cell function and immunotherapy responsiveness. Together, these findings suggest that PRELID1 expression and IL‐7 production in Bgc cells may serve as indicators of an immune‐permissive niche that supports effective CD8^+^ T cell responses and responsiveness to immunotherapy.

A limitation of this study is that the predictive value of Bgc‐associated features for immunotherapy response was primarily inferred from publicly available datasets and experimental models. Future validation in independent external clinical cohorts will be required to fully establish their robustness and translational applicability.

## 4 Conclusions

In conclusion, this study identifies the PRELID1‐VDAC3‐IL‐7 axis in senescence‐like germinal center B cells as a promising therapeutic target to modulate antitumor immunity in colorectal cancer. Therapeutic targeting of this axis holds significant clinical potential to overcome immune resistance, reshape the tumor microenvironment, and enhance the efficacy of current immunotherapies.

## 5 Experimental Section

### 5.1 Patient Samples

This study was approved by the Institutional Review Board of Nanfang Hospital of Southern Medical University. We collected samples from CRC patients who underwent ICIs therapy prior to surgery at Nanfang Hospital between September 2019 and September 2023 (*n* = 45). According to the grade of postoperative tumor regression (TRG), the patients were divided into pCR (*n* = 20) and non‐pCR (*n* = 25) groups. The CAP‐TRG grading system was employed to assess the efficacy of ICIs in CRC. It categorizes tumor response as follows: TRG0 indicates a complete response with no viable tumor cells; TRG1 denotes a partial response with only single cells or small clusters of residual tumor cells; TRG2 suggests a minimal response, characterized by residual tumor cells outnumbering fibrotic tissue; and TRG3 reflects a poor response, with minimal or no tumor cell death and widespread residual cancer [45]. For classification purposes, patients were divided into pCR and non‐pCR groups based on this system, where TRG 1–3 were grouped as non‐pCR and TRG 0 as pCR.

### 5.2 Cell Lines and Cell Culture

GM12878 (RRID:CVCL_7526) were purchased from Meisen CTCC (Hangzhou, China). Engineered cell line 293T was purchased from the Cell Bank of Type Culture Collection of the Chinese Academy of Sciences (Shanghai, China). All cell lines were cultured in RPMI 1640 medium (KeyGEN BioTECH, Jiangsu, China) supplemented with 10% fetal bovine serum (FBS) (Gibco‐BRL, Invitrogen, Paisley, UK). Cells were maintained in a humidified atmosphere containing 5% CO2 at 37°C. All cell lines used in this study tested negative for mycoplasma and were authenticated by short tandem repeat (STR) profiling within four years. All cell line experiments were performed within six months of thawing or cell collection. Cells were transfected with plasmid vectors using Lipofectamine 3000 reagent (Thermo Fisher Scientific, USA) according to the manufacturer's protocol.

### 5.3 Mice

MuMt^−/−^ (C57BL/6J) and WT mice were purchased from Zhaoqing Huaxia Kaiqi Biotechnology. In this mouse model, exon 2 was targeted for deletion using gRNA sequences GTGTTCGTCCCACCACGGGA and CAGCCAGTCGATTTCAGAGA, resulting in a 197 bp deletion fragment. The PCR primers for Ighm‐KO genotyping in MuMt^−/−^ mice are listed in Supplementary Table S1, and the genotyping results are shown in Figure S5.

All animal experiments in this study have been approved by the animal ethics committee of the Tenth Affiliated Hospital of Southern Medical University (approval number: IACUC‐AWEC‐202506019) and conducted under SPF conditions in accordance with approved protocols. The tumor size was measured by vernier caliper twice per week, and the tumor volume (mm^3^) was calculated by the following equation: tumor volume = length × (width)^2^/2.

### 5.4 Isolation of Splenic and Tumor‐Derived Cells

To prepare single‐cell suspensions, mouse spleens and tumors were harvested in DMEM supplemented with 10% FBS. Tumor tissues were minced and digested in DMEM containing 0.5 mg/mL collagenase IV and 0.1 mg/mL DNase I (both from Sigma‐Aldrich) at 37°C for 1 h with shaking, followed by mechanical dissociation on frosted glass slides. The resulting suspensions were filtered through a 70 µm cell strainer (Solarbio Life Science). This method is suitable for subsequent CD8^+^ T cell cytokine detection and exhaustion marker analysis, depending on the experimental needs.

Spleens were harvested and mechanically dissociated in the same manner as tumor tissues, but without enzymatic digestion. The resulting suspensions were filtered through a 70 µm cell strainer. Red blood cells in spleen samples were lysed using 2 mL RBC lysis buffer (Leagene) for 2 min at room temperature and then neutralized with DMEM. The splenocytes were subsequently used for CD8+ T cell sorting.

### 5.5 Flow Cytometry and Cell Sorting

Immune cells were resuspended in staining buffer and pre‐incubated with Fc block (BioLegend) for 10 min. Surface markers were then labeled with specific antibodies for 30 min at 4°C in the dark. For intracellular staining, cells were fixed and permeabilized using Fixation/Permeabilization Concentrate and Diluent (eBioscience, 1:3 ratio) for 30 min, followed by two washes with 1× Permeabilization Buffer. Intracellular antibodies were then added and incubated under the same conditions. Finally, cells were analyzed on a BD flow cytometer. For cytokine detection, cells were stimulated with a cell activation cocktail containing brefeldin A (BioLegend) for 4–6 h prior to staining.

CD8+ T cells were isolated using a mouse CD8+ T‐cell isolation kit (STEMCELL) via MACS. Bgc cells were isolated using a mouse germinal center B cell isolation kit (Milteny) via MACS.

### 5.6 Co‐Culture of CD8^+^ T Cells with Bgc Cells

Mouse CD8^+^ T cells were isolated using MACS and cultured in RPMI‐1640 medium supplemented with 10% fetal bovine serum (FBS). To model early functional impairment and exhaustion‐like features of CD8^+^ T cells, rather than terminal exhaustion or primary activation, CD8^+^ T cells were pre‐activated prior to co‐culture. This design was intended to approximate the activation state of tumor‐infiltrating CD8^+^ T cells and to enable comparative assessment of Bgc‐mediated modulation of effector function under cytokine‐limited conditions. To mimic the physiological activation status of tumor‐infiltrating T cells in vivo and to evaluate the modulatory effects of Bgc cells on effector CD8^+^ T‐cell function rather than their primary activation, CD8^+^ T cells were pre‐activated prior to co‐culture. Cells were activated with anti‐CD3 (2 µg/mL) and anti‐CD28 (5 µg/mL) antibodies for 72 h. Bgc cells were isolated by MACS and co‐cultured with activated CD8^+^ T cells at ratios of 1:2, for 48 h. Cytokine production and expression of exhaustion markers in CD8^+^ T cells were subsequently assessed by flow cytometry. Following co‐culture, CD8^+^ T‐cell effector cytokine production and the expression of exhaustion‐associated markers were evaluated by flow cytometry as indicators of functional suppression or rescue, rather than definitive chronic exhaustion.

### 5.7 In Vivo Bgc Cells Adoptive Transfer Experiment

On day 0, 5 × 10^5^ MC38 cells were subcutaneously implanted into C57BL/6 or muMt^−/−^ mice. The following day, spleen‐derived Bgc cells (5 × 10^5^ cells per mouse) were intravenously administered into the tumor‐bearing mice. A second dose of Bgc cells was delivered on day 7 post‐implantation using the same injection protocol.

### 5.8 Tumor Growth and Treatment

For subcutaneous xenograft models, MC38 CRC cells (1 × 10^6^ per mouse) were injected subcutaneously to induce solid tumor formation. After 19 days, mice were humanely euthanized using an overdose of inhaled isoflurane, followed by cervical dislocation as a secondary physical method to confirm death, in strict accordance with the NIH Office of Laboratory Animal Welfare (OLAW) guidelines and the AVMA Guidelines for the Euthanasia of Animals. Subcutaneous tumors were subsequently collected for histological examination.

In certain experiments, anti‐mouse CD8 antibodies (200 µg per mouse, twice weekly) were administered to deplete CD8^+^ T cells, or anti‐mouse PD‐1 antibodies (200 µg per mouse, twice weekly) were used to block PD‐1 signaling. Corresponding isotype control antibodies were administered in experiments involving CD8 or PD‐1 blockade.

### 5.9 Multiplex Immunofluorescence Staining

FFPE tissue sections (4 µm) were deparaffinized, rehydrated, and subjected to antigen retrieval in citrate buffer (pH 6.0) using a pressure cooker. After blocking with 3% BSA for 30 min at room temperature, primary antibodies were sequentially applied, followed by HRP‐conjugated secondary antibodies and TSA fluorophore development (PerkinElmer). Each staining cycle was followed by microwave‐mediated antibody stripping in citrate buffer. Nuclei were counterstained with DAPI, and slides were mounted using antifade medium.

Cells were fixed in 4% paraformaldehyde for 15 min at room temperature, then permeabilized using 0.2% Triton X‐100 for 10 min. Following a 1‐h blocking step with 5% BSA, cells were incubated with primary antibodies overnight at 4°C. After thorough washing, appropriate fluorophore‐conjugated secondary antibodies were applied for 1 h at room temperature in the dark. Nuclei were stained with DAPI, and samples were mounted using an antifade reagent. Fluorescent images were captured using either confocal or structured illumination microscopy.

Images were acquired using a Leica TCS SP8 confocal microscope or the Pannoramic MIDI II slide scanner (3DHISTECH) for whole‐slide imaging.

### 5.10 Spatial Transcriptomics Analysis of PRELID1 and VDAC Family Genes

Spatial transcriptomic data (GSM7089856) from three CRC tissue sections were preprocessed using the standard pipeline, including log‐normalization and scaling. Expression of PRELID1 and VDAC family genes (VDAC1, VDAC2, VDAC3) was mapped across spatial clusters. Co‐expression was defined as the combined relative expression of all four genes within each spatial spot, represented on a continuous 0–1 scale. Heatmaps were generated to depict relative co‐expression levels, with color from low (deep blue) to high (deep red). No arbitrary binary threshold was applied; the heatmap reflects continuous relative expression across spots. Spatial points exceeding the top quantile of normalized expression for all four genes were considered as highly co‐expressing spots for visualization purposes.

### 5.11 Statistical Analysis

All experiments were independently conducted at least three times. Error bars represent mean ± SD. Data were pre‐processed following standard procedures appropriate for each experimental platform, including routine quality control and normalization where applicable, to ensure data consistency and reliability. Sample sizes (n) for each experiment are specified in the corresponding figure legends. Prior to statistical analysis, data were examined for normal distribution and equal variance. For comparisons between two independent groups, unpaired two‐sided Student's *t*‐tests were applied. One‐way analysis of variance (ANOVA) was employed for comparisons among multiple independent groups. Pearson correlation analysis was conducted to evaluate the statistical relevance of associations between the expression levels of two individual genes or gene signatures. Tumor growth data were analyzed using two‐way ANOVA. When ANOVA indicated statistical significance, post hoc multiple‐comparison tests (Tukey's multiple comparisons test) were applied where appropriate. Some studies choose a representative experimental result from independent experiments to present, where independent experiments refer to experiments conducted on different days. Statistical significance was set at *p*<0.05. The statistical figures and graphical elements in this manuscript were created and compiled using Figdraw (https://www.figdraw.com/), BioGD (Phttps://biogdp.com/) and Adobe Illustrator 2023 (Adobe, San Jose, CA, USA). Statistical analyses were performed using GraphPad Prism 9 (GraphPad Software Inc.).

## Author Contributions

Y.L., H.X., X.Z., and S.H. contributed equally to this work. J.Z. and Y.L. designed the experiments. Y.L., H.X., X.Z., and S.H. performed the experiments, drafted the manuscript and revised the manuscript. S.H. contributed to data analysis. L.H., Z.H., J.C., and Y.W. assisted with the animal experiments.

## Conflicts of Interest

The authors declare no conflicts of interest.

## Supporting information




**Supporting File 1**: advs74393‐sup‐0001‐Data.xlsx.

**Supporting File 2**: advs74393‐sup‐0002‐Data.pdf.

**Supporting File 3**: advs74393‐sup‐0003‐SuppMat.docx.

## Data Availability

All source data and materials are included in the Supporting Data Values file. Additional underlying datasets and relevant analytical code used in this study are available from the corresponding author upon reasonable request. Detailed experimental procedures are provided in the supplementary materials.
