# Supplementary figures and images for: PRELID1 and VDAC3 Coordinate a Senescence‐Like State in Germinal Center B Cells to Promote IL‐7–Driven Antitumor Immunity in Colorectal Cancer
Source: Adv Sci (Weinh). 2026 Feb 18;13(23):e21951. doi: 10.1002/advs.202521951 (PMC13104075; doi:10.1002/advs.202521951)

Figure 2K

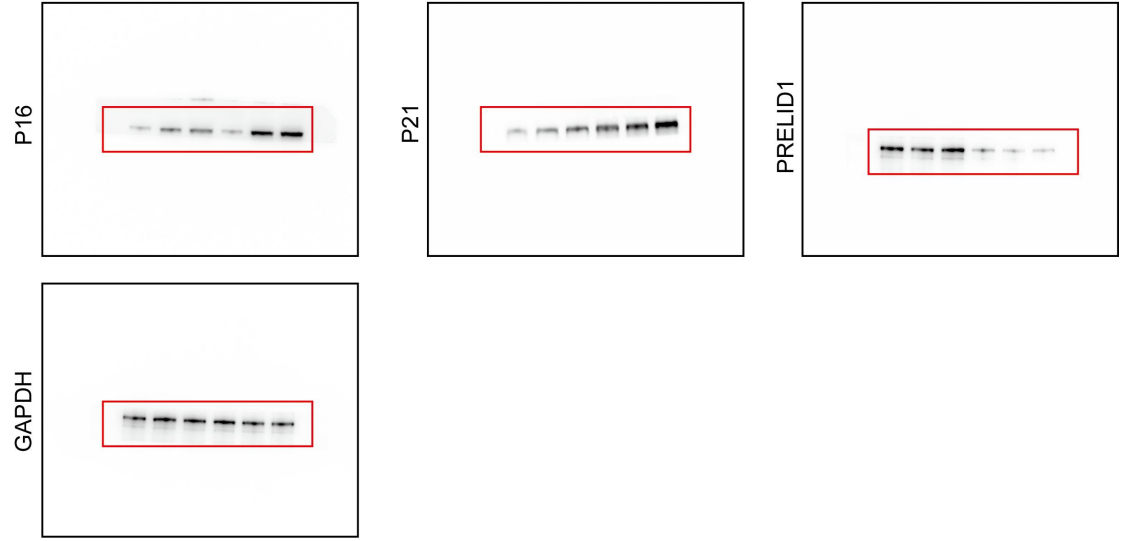

Figure 4A

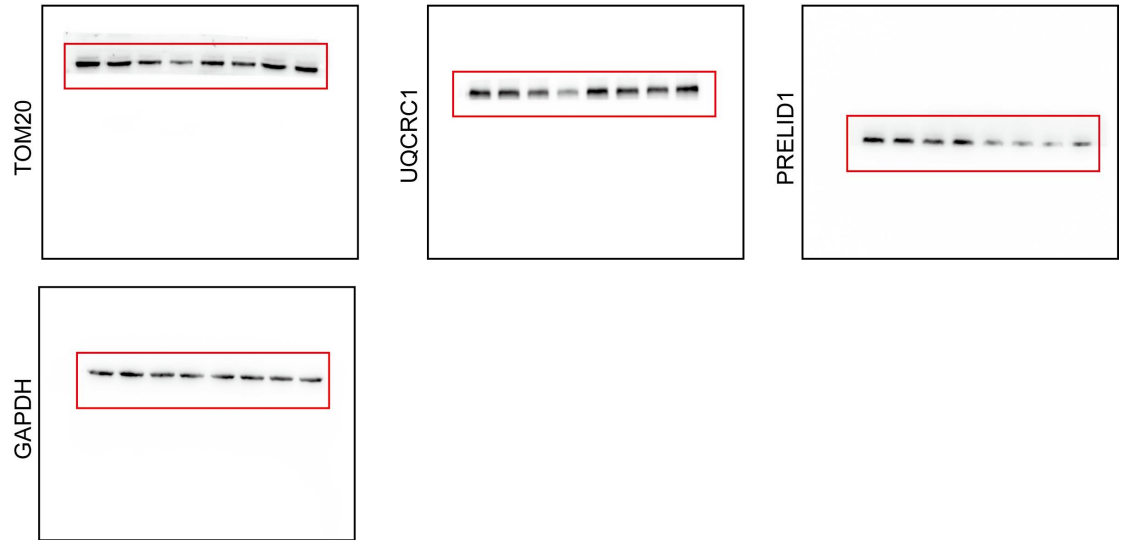

Figure 4B

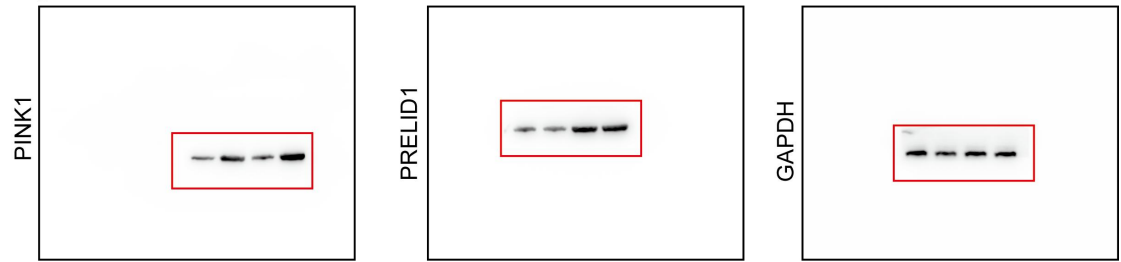

Figure 4H

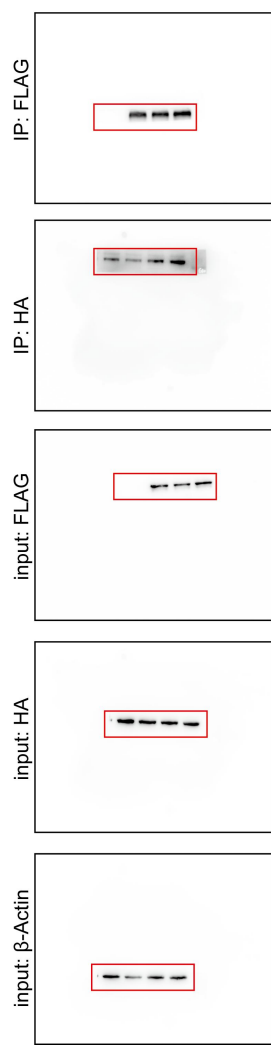

Figure 4I

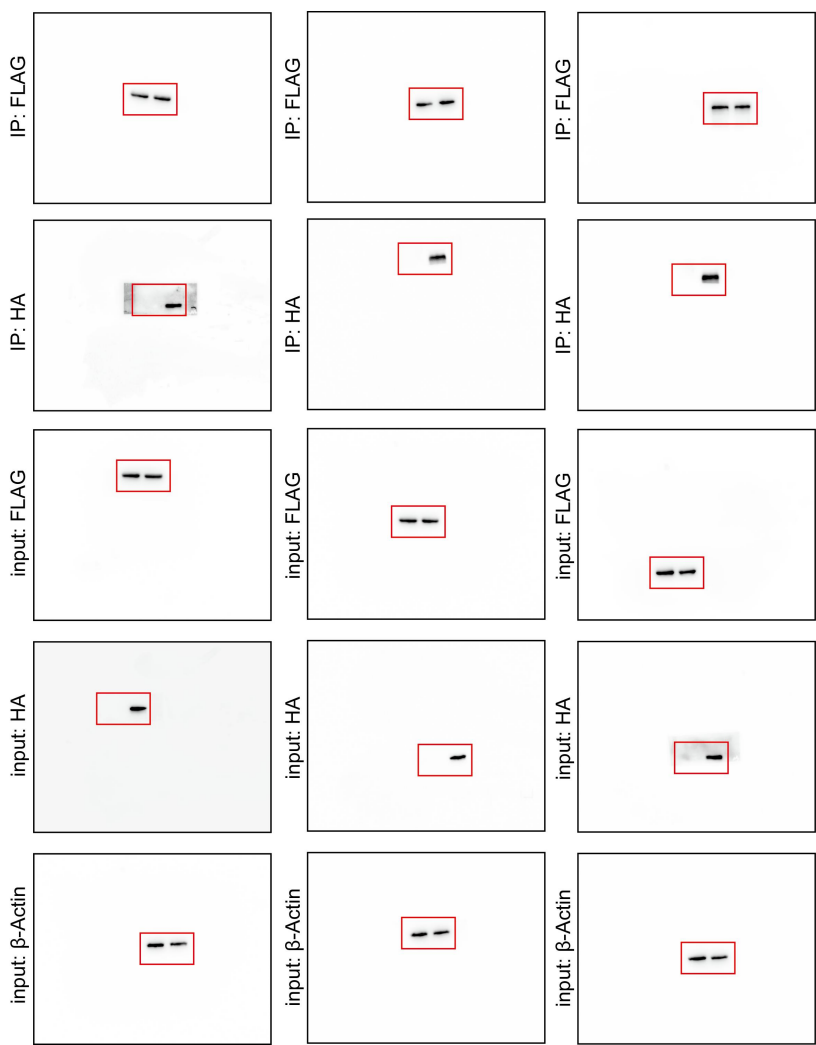

Figure 4J

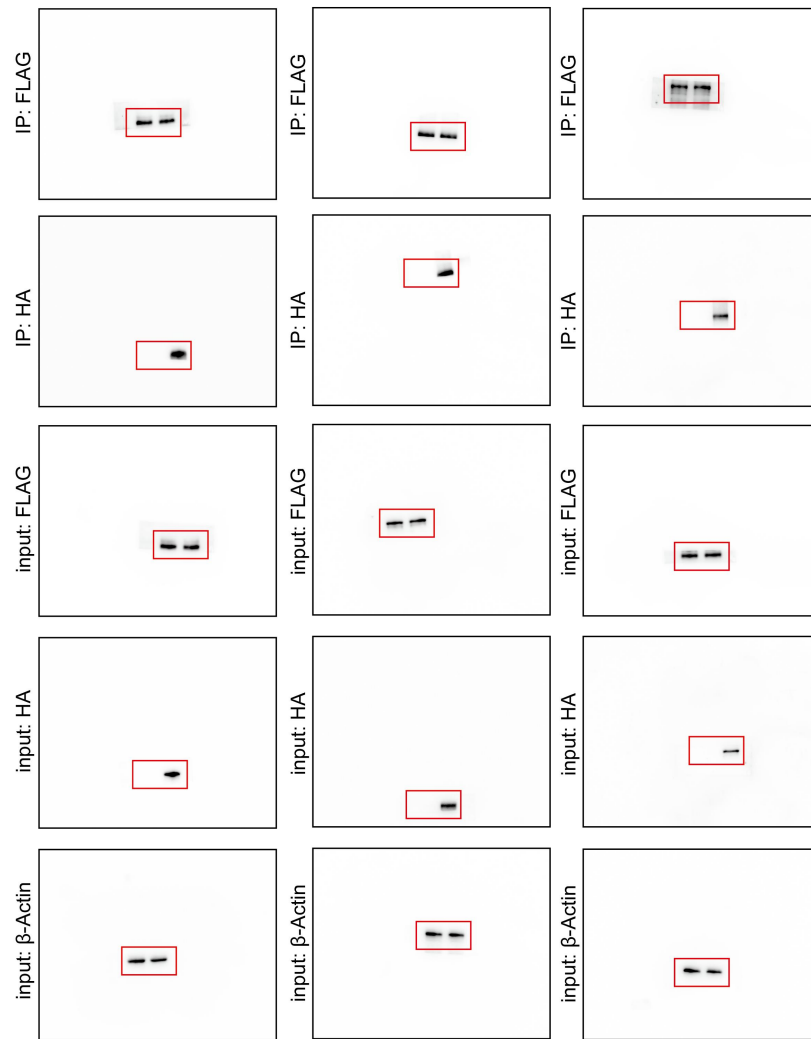

Figure 4K

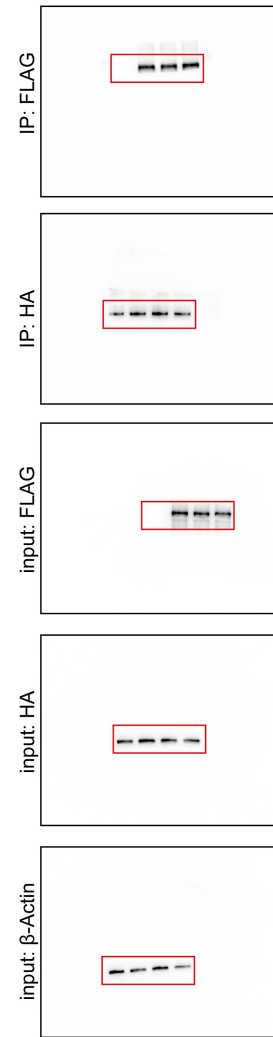

Figure 4L

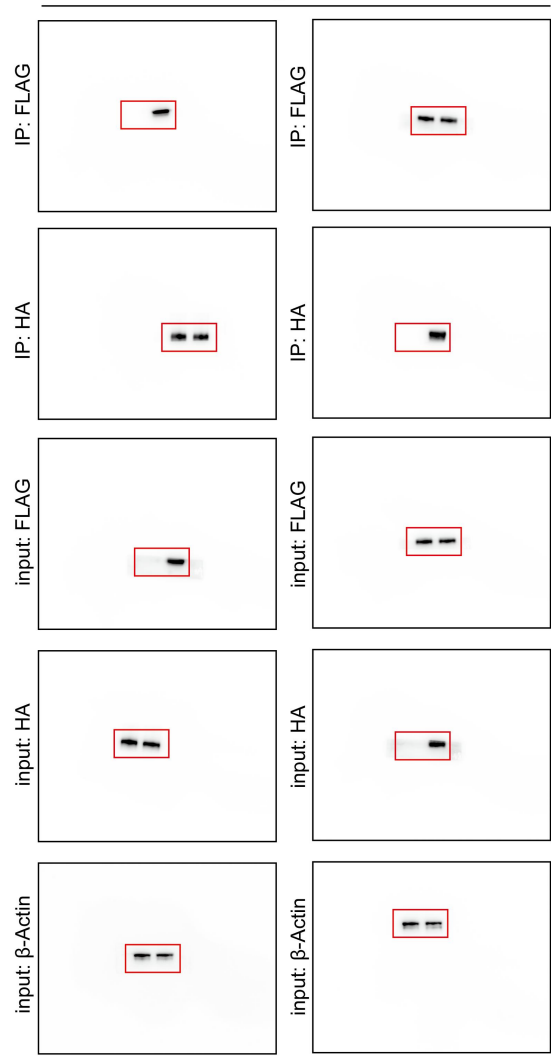

Figure 4M

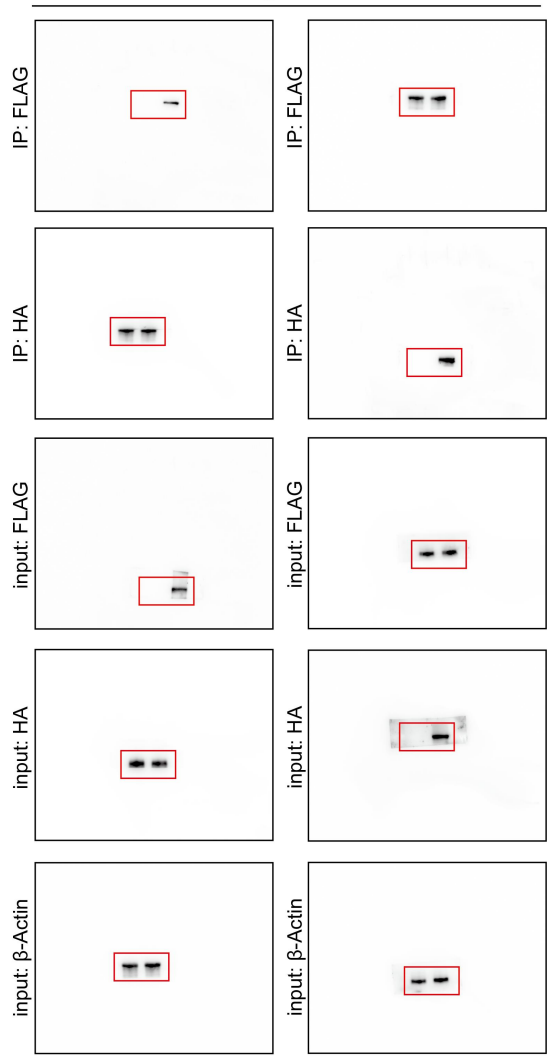

Figure 7B

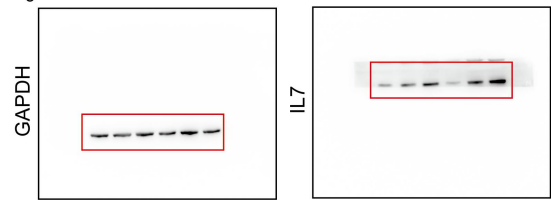

Supplement: Supplementary file 2 — Supporting File 2: advs74393‐sup‐0002‐Data.pdf. [file ADVS-13-e21951-s001.pdf]
