# Supplementary material for: PRELID1 and VDAC3 Coordinate a Senescence‐Like State in Germinal Center B Cells to Promote IL‐7–Driven Antitumor Immunity in Colorectal Cancer
Source: Adv Sci (Weinh). 2026 Feb 18;13(23):e21951. doi: 10.1002/advs.202521951 (PMC13104075; doi:10.1002/advs.202521951)
Supplement: Supplementary file 3 — Supporting File 3: advs74393‐sup‐0003‐SuppMat.docx. [file ADVS-13-e21951-s002.docx]

**Supplementary Materials for**

**PRELID1 and VDAC3 Coordinate a Senescence-like State in Germinal Center B Cells to Promote IL-7–Driven Antitumor Immunity in Colorectal Cancer**

**Correspondence to:** Jun Zhou: 17665772852@163.com

**This PDF file includes:**

**Supplementary Materials and Methods,**

**Figures S1–S15,**

**and Tables S1–S2.Supplementary Materials and Methods**

**Method for scRNA-seq data analysis**

**scRNA-seq dataset and differential expression analysis**

We used the publicly available dataset GSE205506, which includes single-cell RNA sequencing from 19 d-MMR/MSI-H colorectal cancer patients treated with PD-1 blockade. Cases achieving pathological complete response (pCR) and those without (non-pCR) were selected. Raw FASTQ files were processed using the 10x Genomics Cell Ranger pipeline and mapped to GRCh38. Differential gene expression between pCR and non-pCR groups was calculated, with genes considered significant when |log₂ fold change| ≥ 1 and FDR (adjusted P-value) < 0.05. CellChat analysis was subsequently employed to compare cell–cell communication features between groups.

**Cell-cell communication analysis**

CellChat^1^ was used for Inference and analysis of cell-cell communication. Gene expression matrix and cell type labels are exported from Seurat object, then create a CellChat object using “createCellChat”. Cellular communication Modeling, analysis and visualization are conducted by following standard procedures. CellChat (http://www.cellchat.org/), a tool that enables to quantitatively estimate and analyze the cell-cell communication network with scRNA-seq data as the input. Thereafter, it offers capabilities for further data exploration, analysis as well as visualization. Through defining the outgoing and incoming interaction strengths as the out- and in-degree centrality measures in weighted cell-cell communication networks, the remarkable signaling mechanisms and targets were analyzed.

**Gene set enrichment analysis**

Differentially expressed genes were calculated between different disease conditions of cell types via using the Seurat FindMarkers function with default parameter of “MAST” test. “ClusterProfiler” was carried out to detect Gene Ontology (GO) and Kyoto Encyclopedia of Genes and Genomes (KEGG) pathways enriched by differentially expressed^2^. The terms with false discovery rate (FDR) <0.05 were regarded as significant enrichment.

**Identification of marker genes and differential expression genes (DEG)**

To calculate DEG between two group of cells e.g. Treatment vs Control, Seurat FindMarkers function with method “MAST” were applied for two group of cells with parameter “min.pct = 0.01, logfc.threshold = 0.01”.

For marker genes and DEG lists, GO and pathway analyses were performed by R package ClusterProfile (V3.18.1)^3^.

**Construction of Cellular Senescence Score Using GSVA**

The list of senescence-associated genes was obtained from the CellAge database (https://genomics.senescence.info/cells/). The cellular senescence score was calculated using GSVA based on 525 upregulated (Up_CS) and 734 downregulated (Down_CS) senescence-associated genes. Transcriptomic data were analyzed in R (v4.1.1) using the GSVA and GSEABase packages.

**Calculation of CellAge Scores in Single-Cell Transcriptomic Data**

For each cell within the single-cell RNA sequencing (scRNA-seq) dataset, a corresponding CellAge Scores was computed. Given the variability in the number of detectable genes across individual cells, we first identified the intersection between the expressed genes in each cell and the predefined gene set associated with cellular senescence. The CellAge Scores was then calculated based on this intersection, following a principle similar to that described previously. The scoring was performed using the R package escape, with the function enrichIt applied for enrichment analysis.

Cells were ranked based on their cellular senescence scores in ascending order. The top 5% as the CellAge high group.

**SASP Scoring in B Cells**

To avoid bias from differential gene selection, all Senescence-associated secretory phenotype (SASP) - related genes were used to score B cells. The AddModuleScore function in the Seurat R package was applied, and cells with higher scores were considered to exhibit stronger SASP-like features. The SASP gene set was obtained from the Senescence-Associated Secretory Phenotype (SASP) pathway in the Reactome database (https://reactome.org/), comprising a total of 75 genes.

**METHOD DETAILS**

**Primers, shRNAs**

qPCR primers and shRNAs are shown in [supplemental table S1](https://jitc.bmj.com/content/10/2/e003663.long#DC1).

**Plasmid construction and lenti-virus transduction**

All lentiviral vectors and control vectors were purchased from Tsingke ( Guangzhou, China). To generate lentiviral supernatant, the plasmids containing the genetic information of the lentivirus, as well as the packaging vector psPAX2 (Addgene, 12260) and envelope vector PMD2.G (Addgene, 12259), are co-transfected into 293T cells. After 12 h of transfection, the medium was replaced, and the virus-containing supernatant was collected 48 h later. The collected supernatant was filtered through a 0.45 µm PVDF filter with small pores (Millipore, Darmstadt, Germany). And then, 8 µg/mL Polybrene (Sigma-Aldrich, 107689), a transduction enhancer, was added to the filtered supernatant prior to infection of the target cells.

**Immunohistochemistry (IHC)**

Immunohistochemistry was performed as previously described^4^, to investigate the expression of proteins in clinical specimens and mouse subcutaneous tumor tissue. The tissue sections were incubated overnight using primary antibodies (Primary antibodies used are listed in [supplemental table S2](https://jitc.bmj.com/content/10/2/e003663.long#DC1)). Mayer’s haematoxylin was used for nuclear counterstaining. For IHC staining, we used a semi-quantitative method, which was calculated by an established semi-quantitative assessment of both the intensity of staining and the percentage of positive cells following an established procedure. The staining intensity in the malignant cell was scored as 0, 1, 2, or 3 for the presence of negative, weak, intermediate, or strong staining, respectively. As well as, the expression ratio was scored as 1 (0-25%), 2 (26-50%), 3 (51-75%), and 4 (76-100%). The final score for immunohistochemistry is staining intensity multiplied by area, for a total of 12 points. In this study, we define a score greater than or equal to 6 as a “high expression”, and a score less than 6 as a “low expression”. The slides were reviewed and scored by at least two, and usually three, certified anatomic pathologists. The 5% of discrepancies were resolved through simultaneous re-evaluation. The Pearson’s chi-squared test (χ^2^) was used to determine the significance of the correlation.
**Western Blot**

Total protein extraction was performed with RIPA lysis buffer supplemented with a protease inhibitor cocktail. And protein was quantified by the BCA Protein Assay Kit (Pierce, KeyGEN BioTECH, Jiangsu, China). After denaturation, proteins were separated by SDS-PAGE gel and transferred to the PVDF membrane (Millipore, Darmstadt, Germany). A Tris buffer containing 0.1% Tween-20 and 5% non-fat dry milk was used to block the membrane at RT for 1 h. The membrane was incubated overnight with antibodies (Primary antibodies used are listed in [supplemental table S2](https://jitc.bmj.com/content/10/2/e003663.long#DC1).).Blots were then washed and incubated for 1 h at RT with horseradish peroxidase (HRP)-labeled secondary antibodies (anti-rabbit IgG or anti-mouse IgG, Cell Signaling Technology, 1: 10,000). The protein was detected by an ECL chemiluminescence solution (Epizyme Biomedical Technology Co., Ltd, Shanghai, China) and finally visualized using an enhanced chemiluminescence detection system (Tennon5200, Shanghai, China).

**Co-immunoprecipitation (Co-IP) Assay**

Cells were lysed in a non-denaturing lysis buffer containing protease and phosphatase inhibitors. The lysates were pre-cleared with control IgG and protein A/G agarose beads, then incubated with specific primary antibodies overnight at 4°C with gentle rotation. Protein complexes were captured using protein A/G beads, followed by extensive washing with lysis buffer to remove non-specific interactions. Bound proteins were eluted by boiling in SDS-PAGE loading buffer and analyzed by Western blotting.

**RNA isolation, reverse transcription, and quantitative PCR (qPCR)**

Total RNA was extracted from cells and tissue using the Trizol reagent (Invitrogen, Carlsbad, California). And then 1 µg RNA was used to reverse transcribe cDNA using the Evo M-MLV Reverse Transcription Kit (Accurate Biotechnology Co., Ltd, Hunan, China). QPCR analyses were carried out on retrotranscribed cDNAs with the SYBR® Geen Pro Taq HS qPCR kit (Accurate Biotechnology Co., Ltd, Hunan, China). Expression was measured on an Applied Biosystems 7500 Fast Real-Time PCR system. Experiments were performed at least three times, with duplicate replicates. The quantification is based on the 2^-ΔΔCt^ method using the housekeeping gene GAPDH as a normalizer.


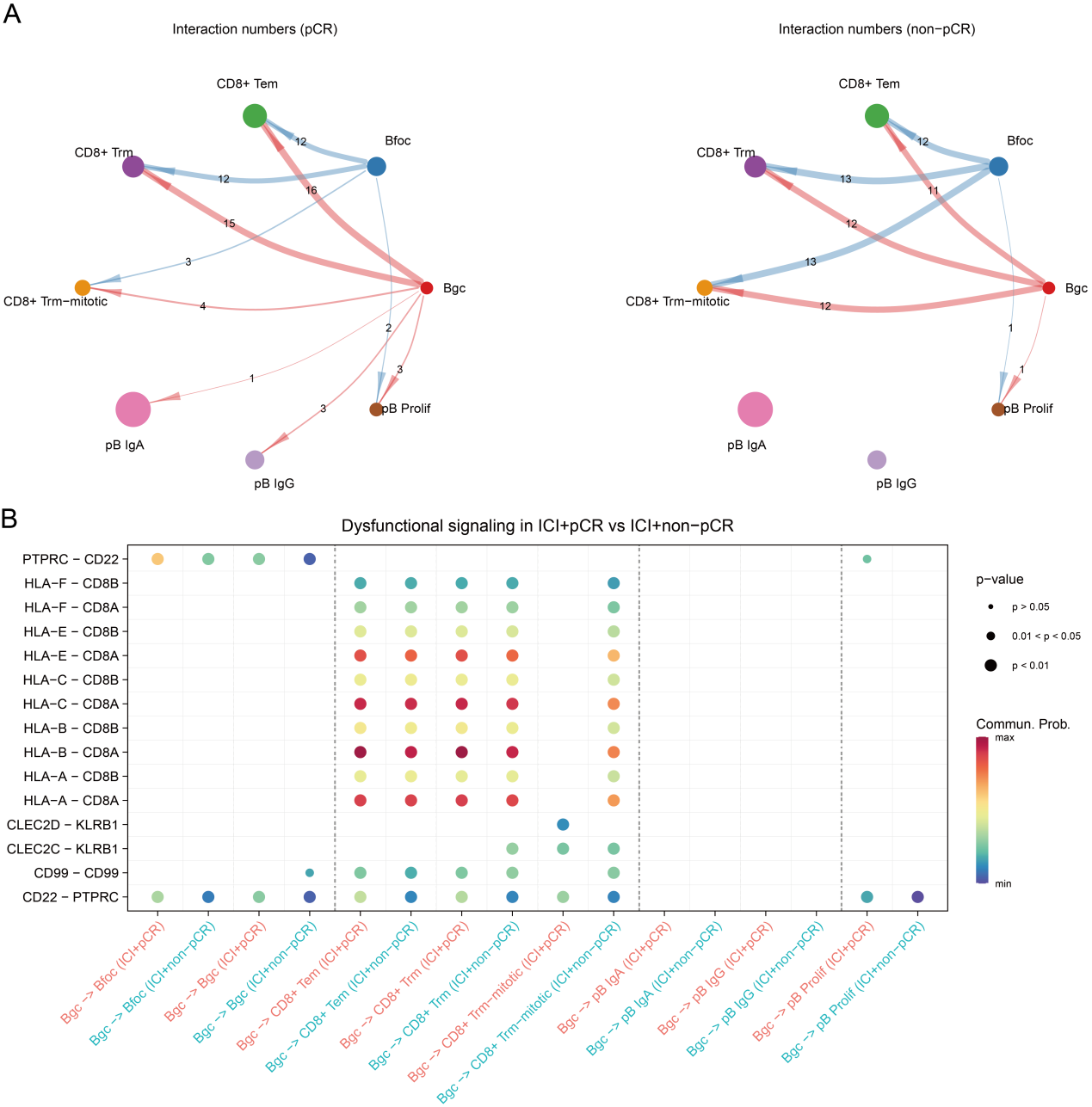


**Figure S1. (A)** Quantitative analysis of cell–cell interaction number in pCR and non-pCR samples. **(B)** Quantitative visualization of ligand–receptor interaction strength between B and T cell populations in pCR and non-pCR samples. Each dot represents a ligand–receptor pair, with dot size and color indicating interaction probability and strength, respectively.


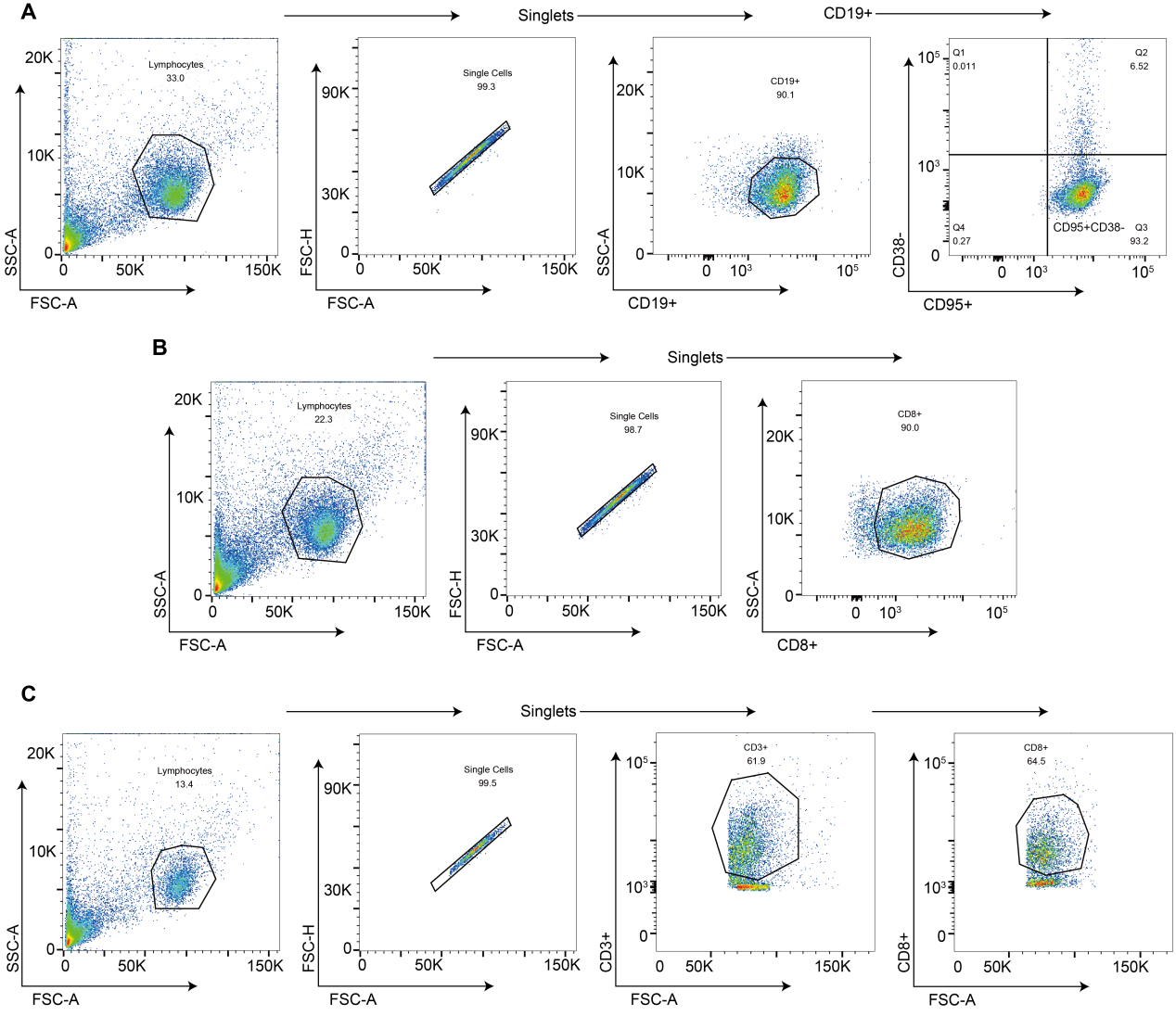


**Figure S2.** Purity validation and gating strategies for flow cytometric analysis of primary Bgc cells **(A)**, primary CD8⁺ T cells **(B)**, and CD8⁺ T cells after co-culture with Bgc cells **(C)**.


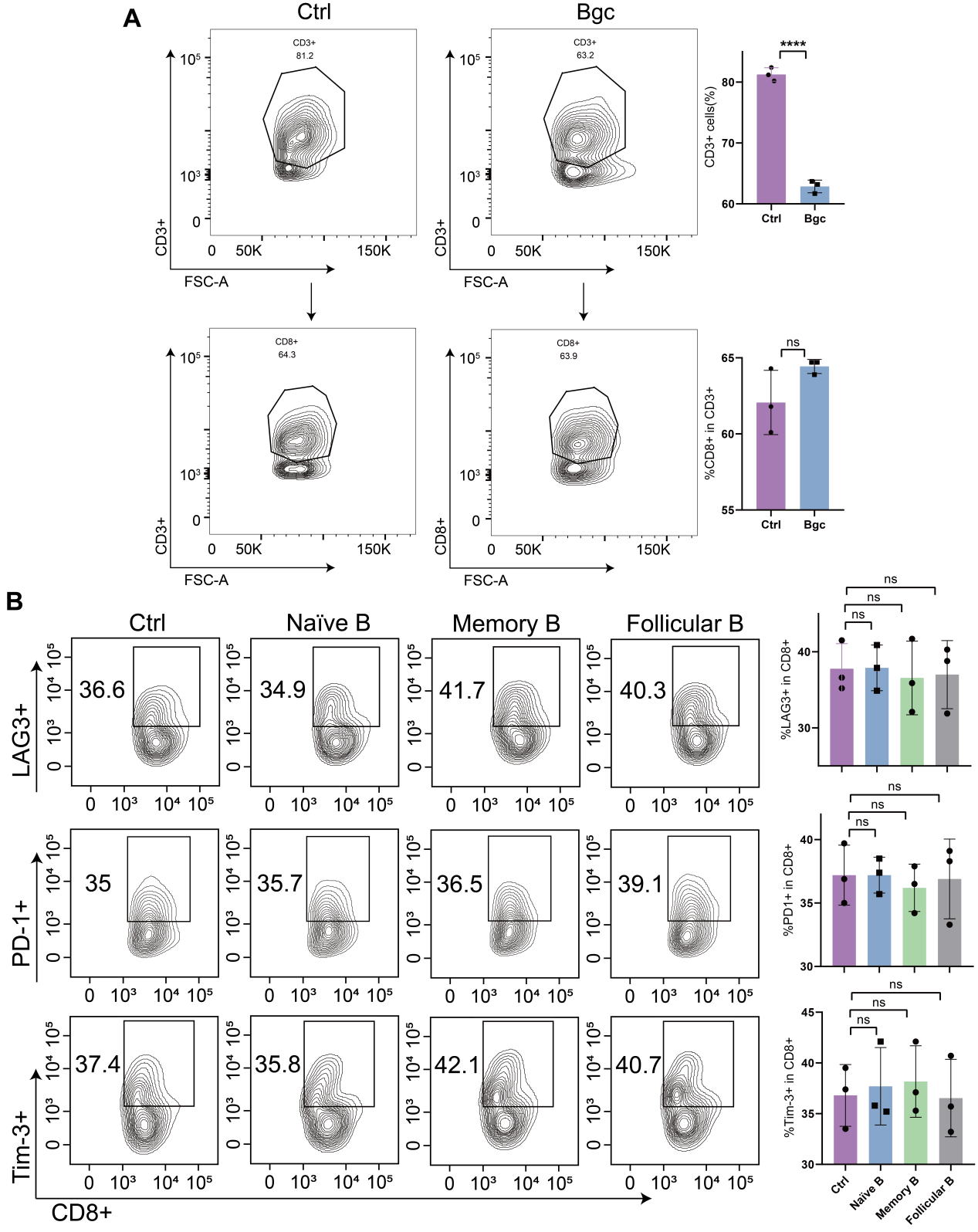


**Figure S3. (A)** Representative flow-cytometry plots showing CD3⁺ and CD8⁺ T-cell proportions in a sample before and after co-culture with Bgc cells. **(B)** Flow-cytometry analysis of CD8⁺ T cells following co-culture with germinal-center (GC) B cells, naïve B cells, memory B cells, or follicular B cells.

**
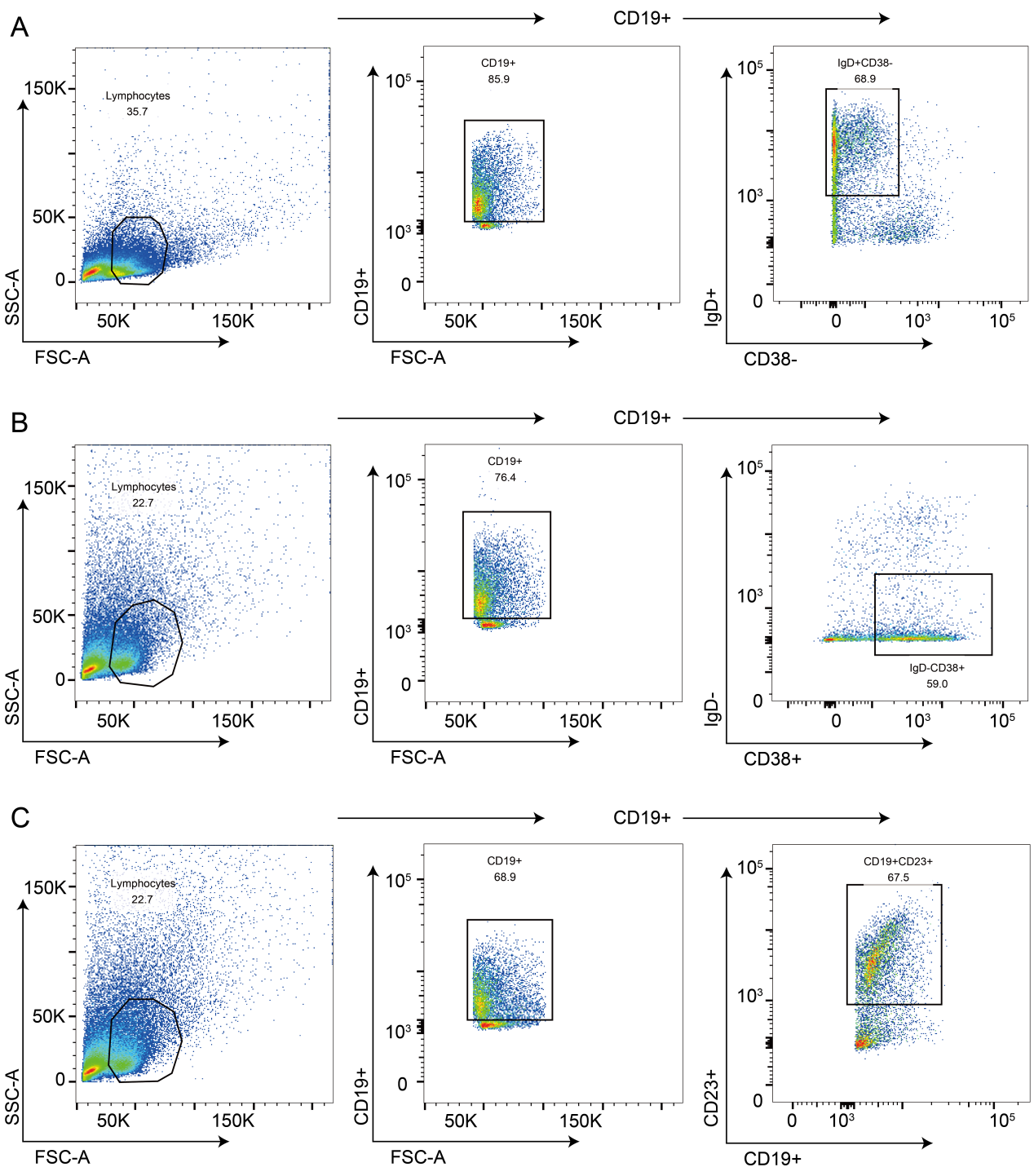
**

**Figure S4.** Representative flow-cytometry plots showing the gating strategy for B-cell subsets: naïve B cells (CD19⁺ IgD⁺ CD38⁻) in **(A)**, memory B cells (CD19⁺ IgD⁻ CD38⁺) in **(B)**, and follicular B cells (CD19⁺ CD23⁺) in **(C)**.

**
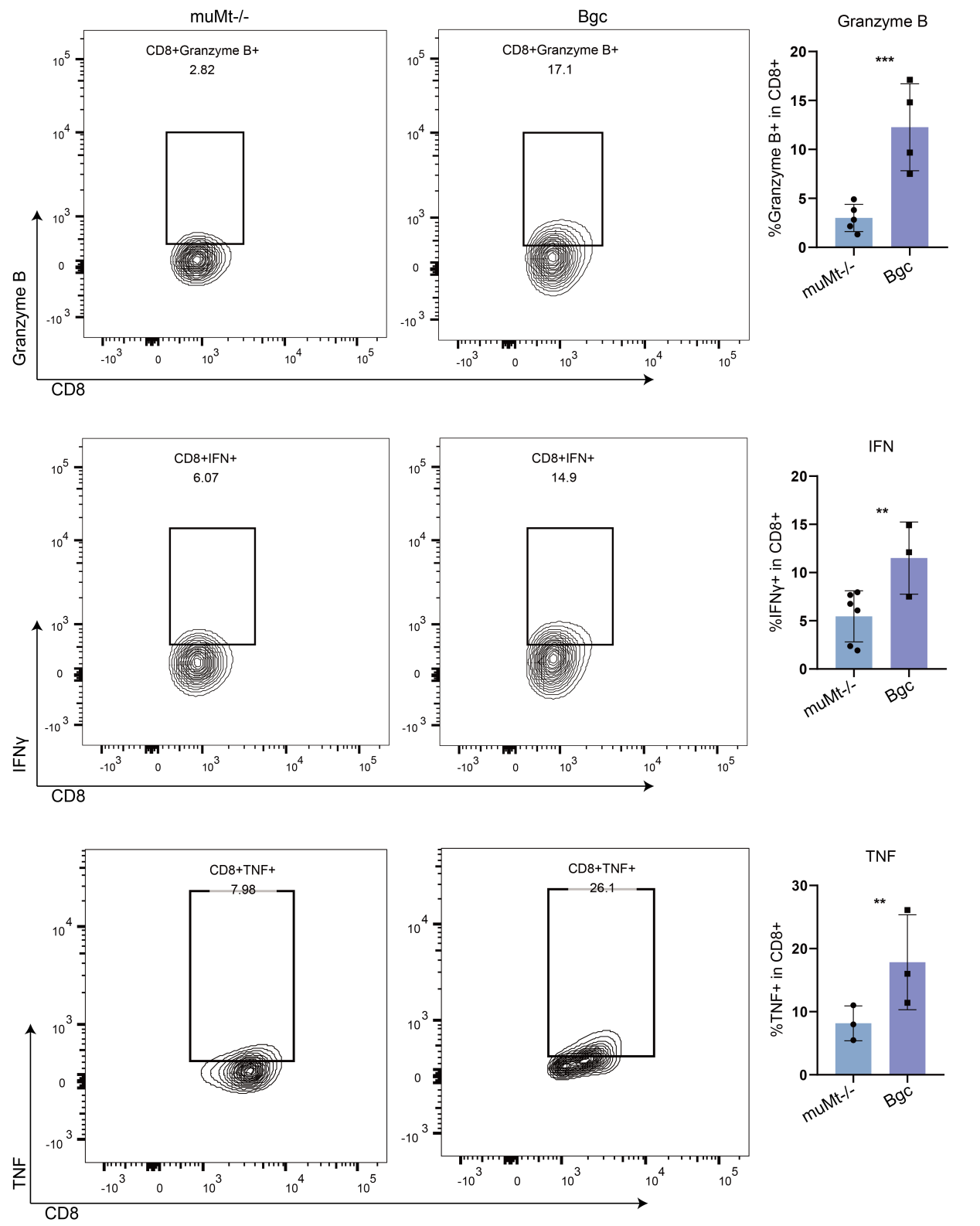
**

**Figure S5.** Flow cytometric analysis of tumor-infiltrating lymphocytes from muMt⁻/⁻ mice with or without adoptive transfer of Bgc cells. Tumors were harvested and analyzed for CD8⁺ T cell expression of effector function markers.


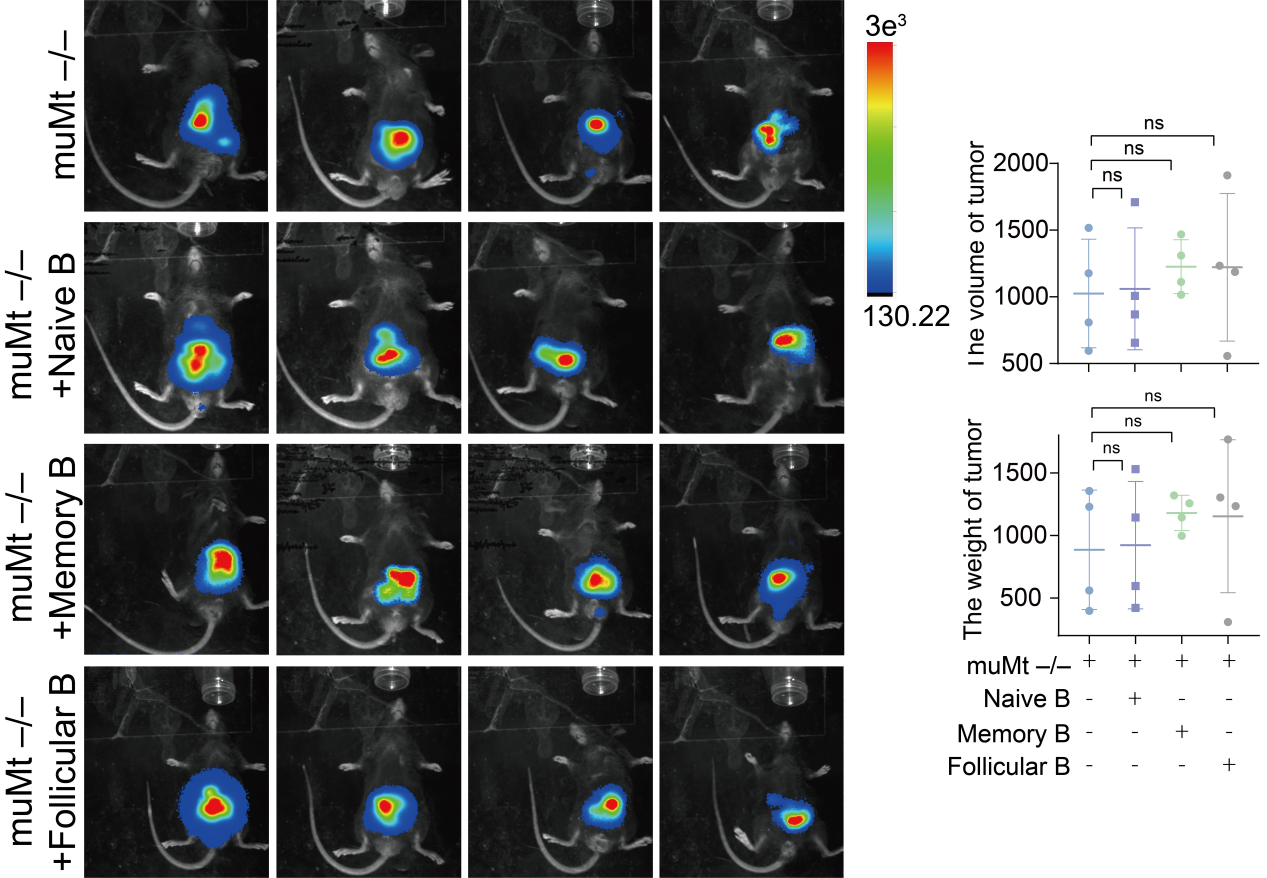


**Figure S6.** Representative bioluminescence imaging, tumor volumes, and tumor weights in mice with or without transfer of naïve B cells, memory B cells, or follicular B cells. I.p., intraperitoneal injection; n = 4 mice per group; data presented as mean ± SEM.


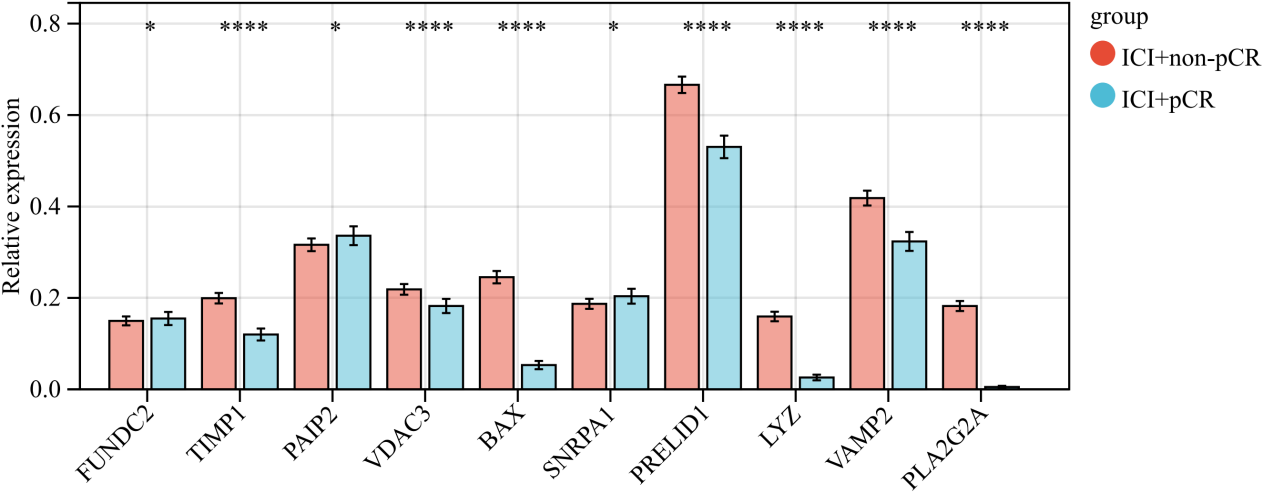


**Figure S7.** Bar graphs show the relative expression of the ten proteins identified from differential gene expression analysis based on log fold change (logFC). Data are presented as mean ± SEM for each group, with statistical significance assessed using two-sided Student’s t-test.

**
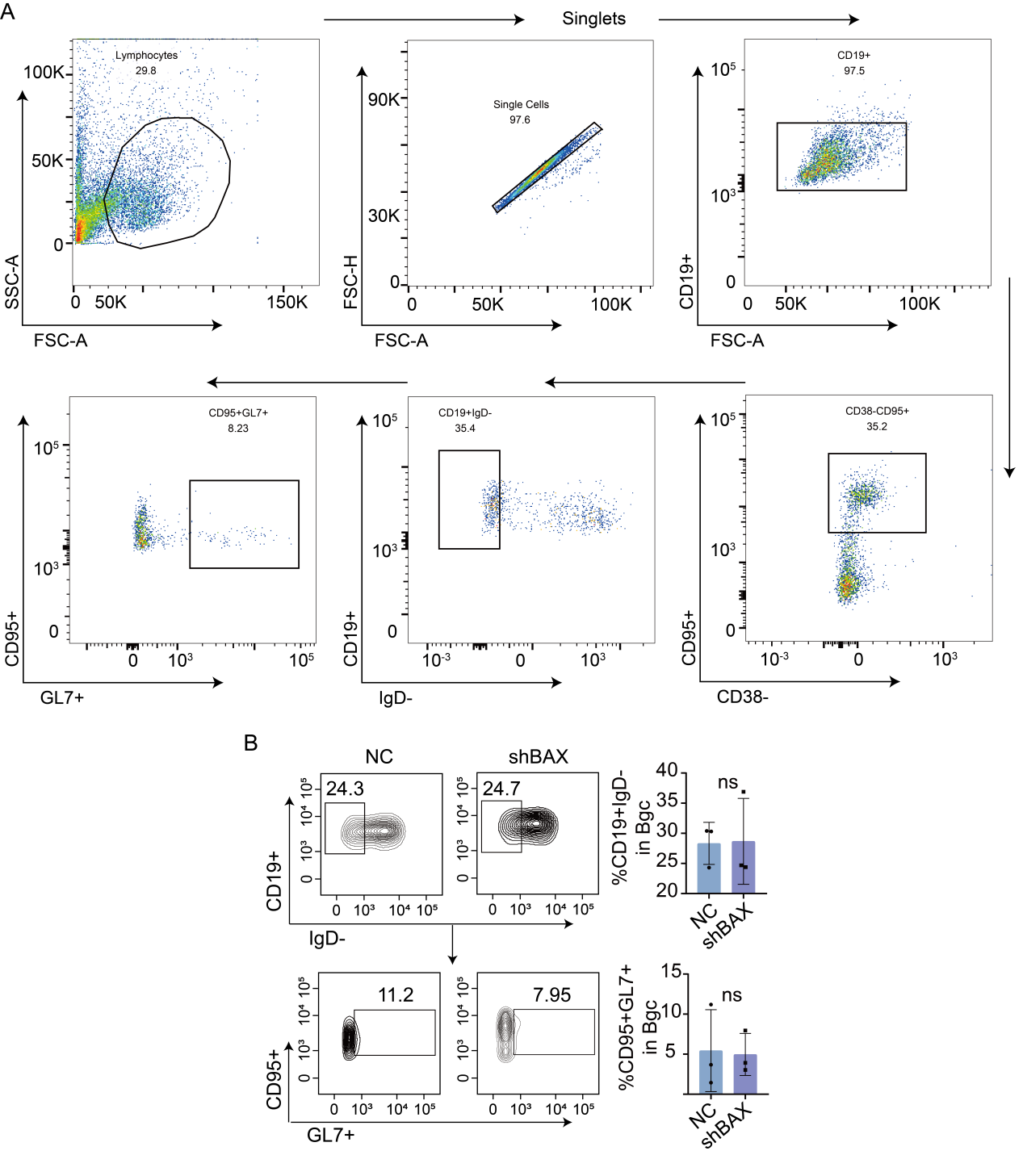
**

**Figure S8. (A)** Representative flow-cytometry plots showing the gating strategy for IgD^lo^- activated and GL7^+^ CD95^+^ B cells. **(B)** Flow cytometry was used to evaluate Bgc cell responses under different treatment conditions.

**
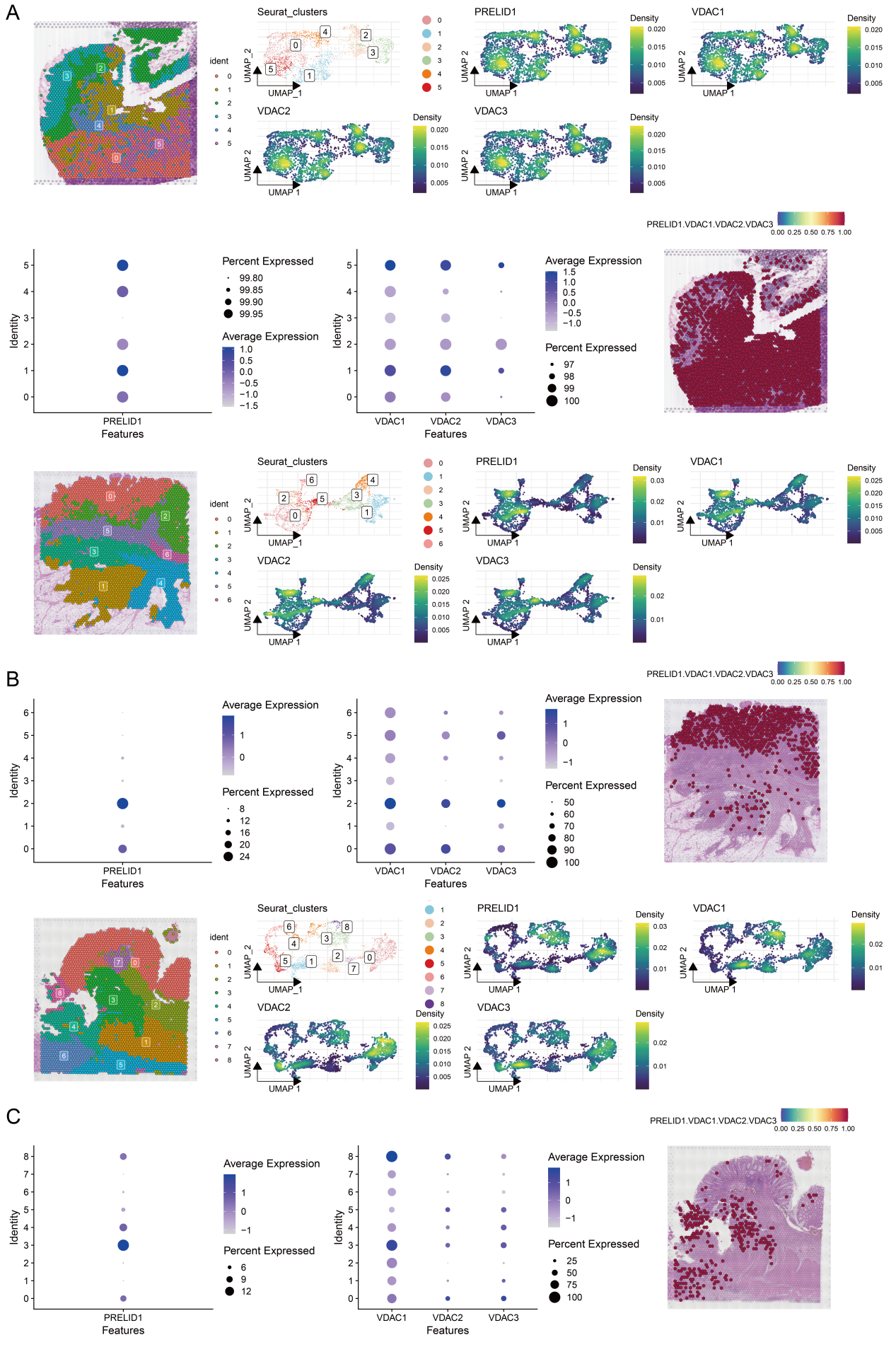
**

**Figure S9.** Analysis identified six major spatial clusters across three CRC tissue sections. Expression of PRELID1 and VDAC family genes (VDAC1, VDAC2, VDAC3) is shown across clusters, and co-expression of all four genes is indicated for spatial points within the tissue.

**
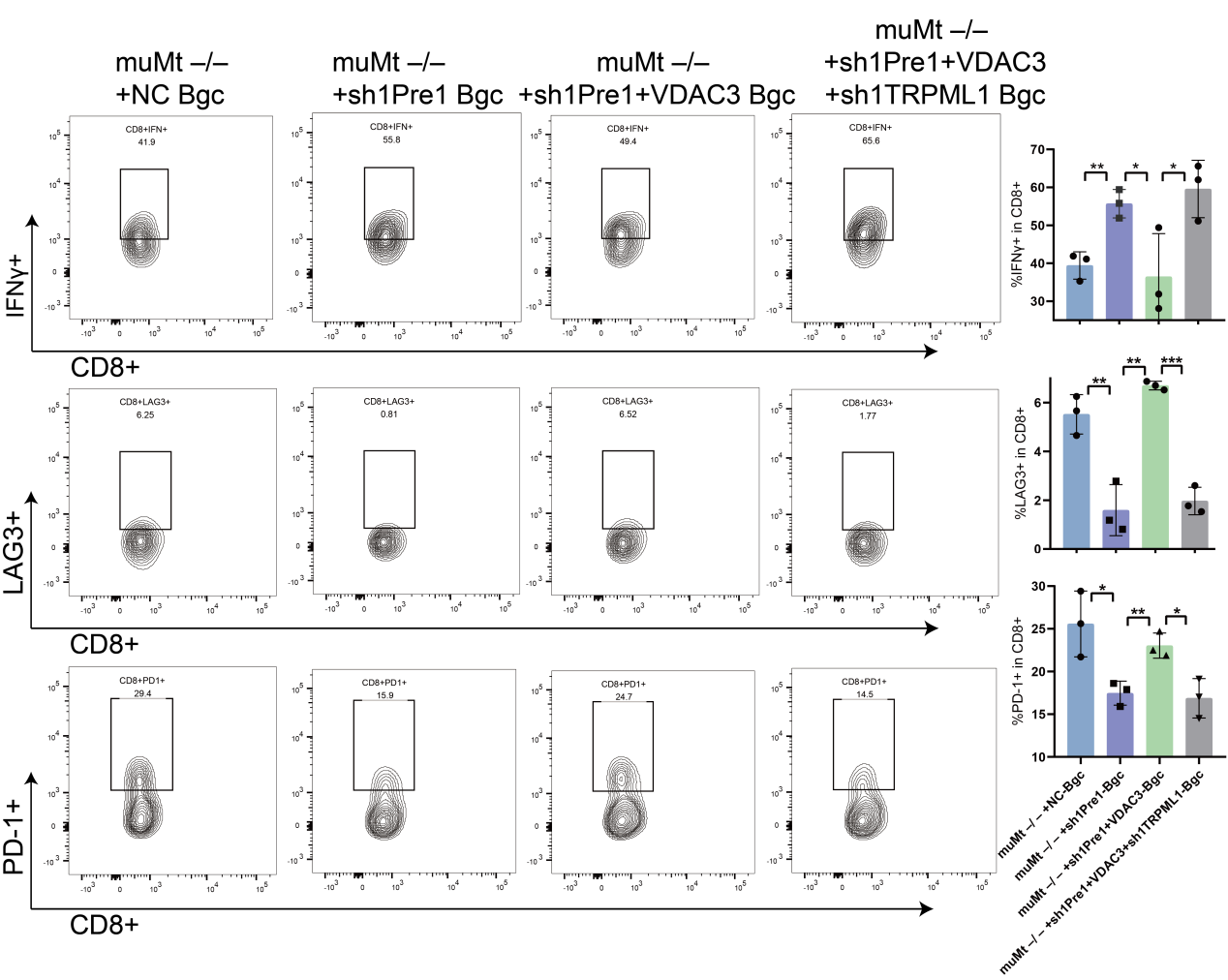
**

**Figure S10.** Flow cytometry analysis of exhaustion markers (PD-1, LAG3, and Tim-3) on CD8^+^ T cells.


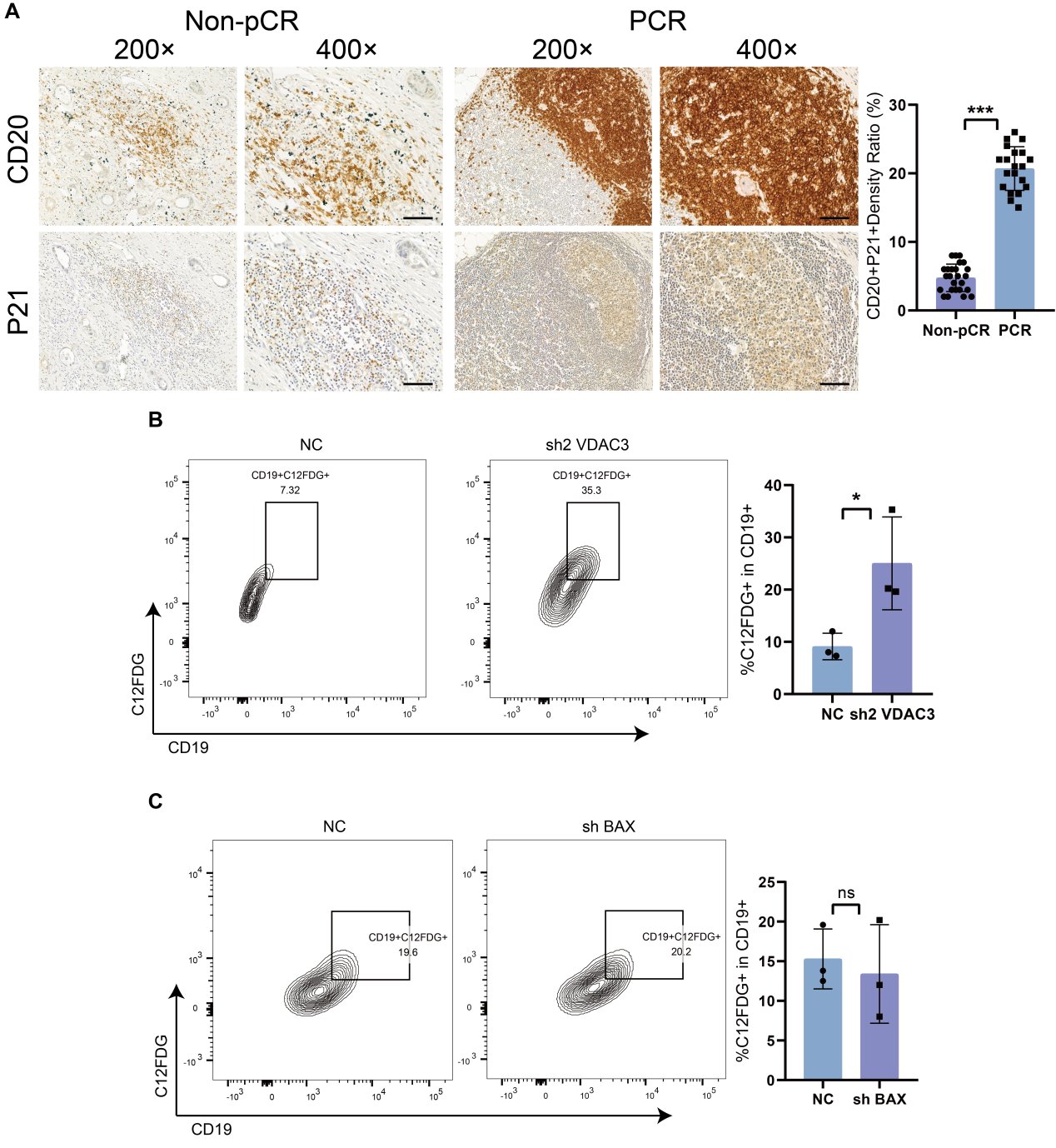


**Figure S11. (A)** Immunohistochemical analysis was performed to assess the expression of P21 in CD20^+^ cells within colorectal cancer tissues from pCR and non-pCR patients. Scale bars for 20× figures are 100 μm, and those for 40× figures are 50 μm. **(B-C)** Flow cytometry was used to evaluate the senescence level of Bgc cells under different treatment conditions.


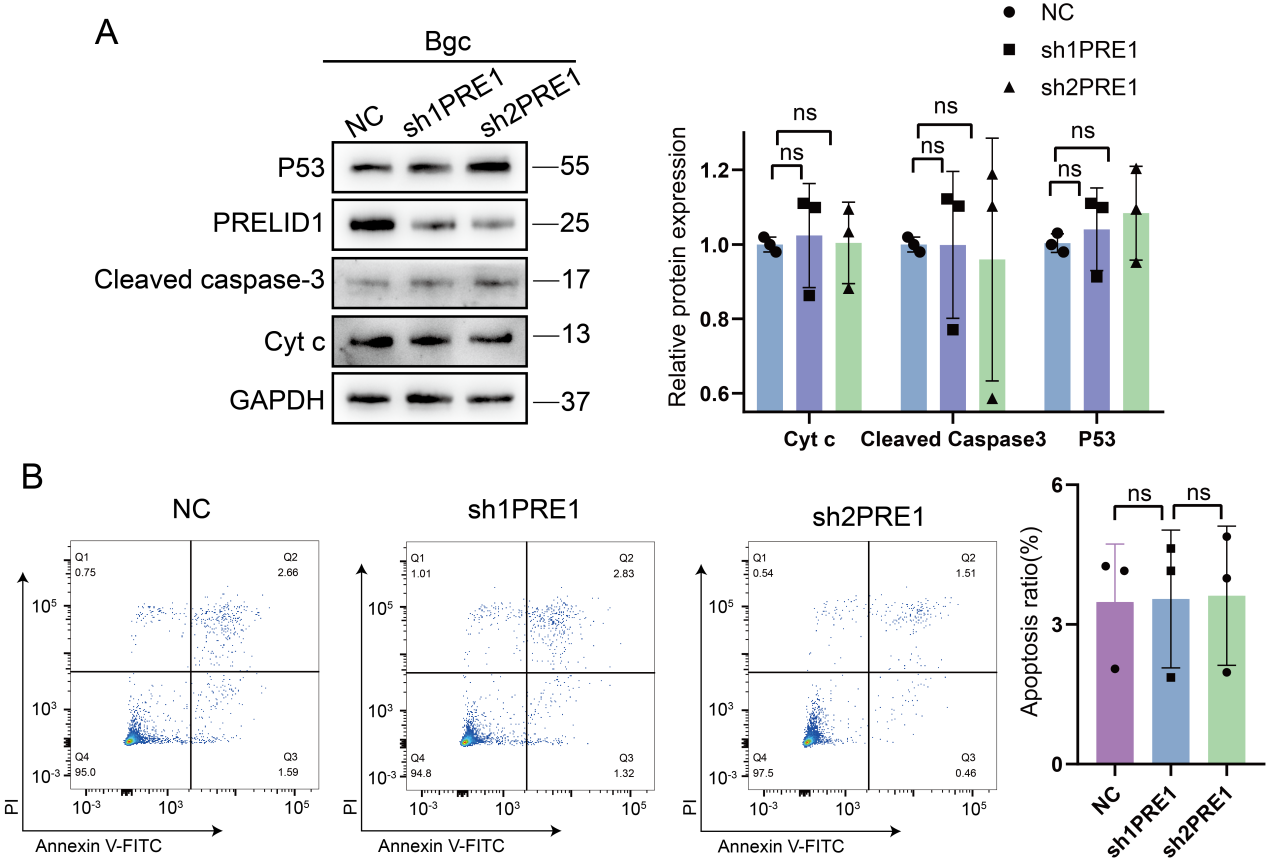


**Figure S12.** (A) Western blot analysis of apoptosis markers P53, cleaved caspase-3, and cytochrome c in control and PRELID1 knockdown cells. (B) Annexin V/PI staining of control and PRELID1 knockdown cells. Data represent n = 3 independent experiments per group and are presented as mean ± SEM, with statistical comparisons performed using two-sided Student’s t-test.

**
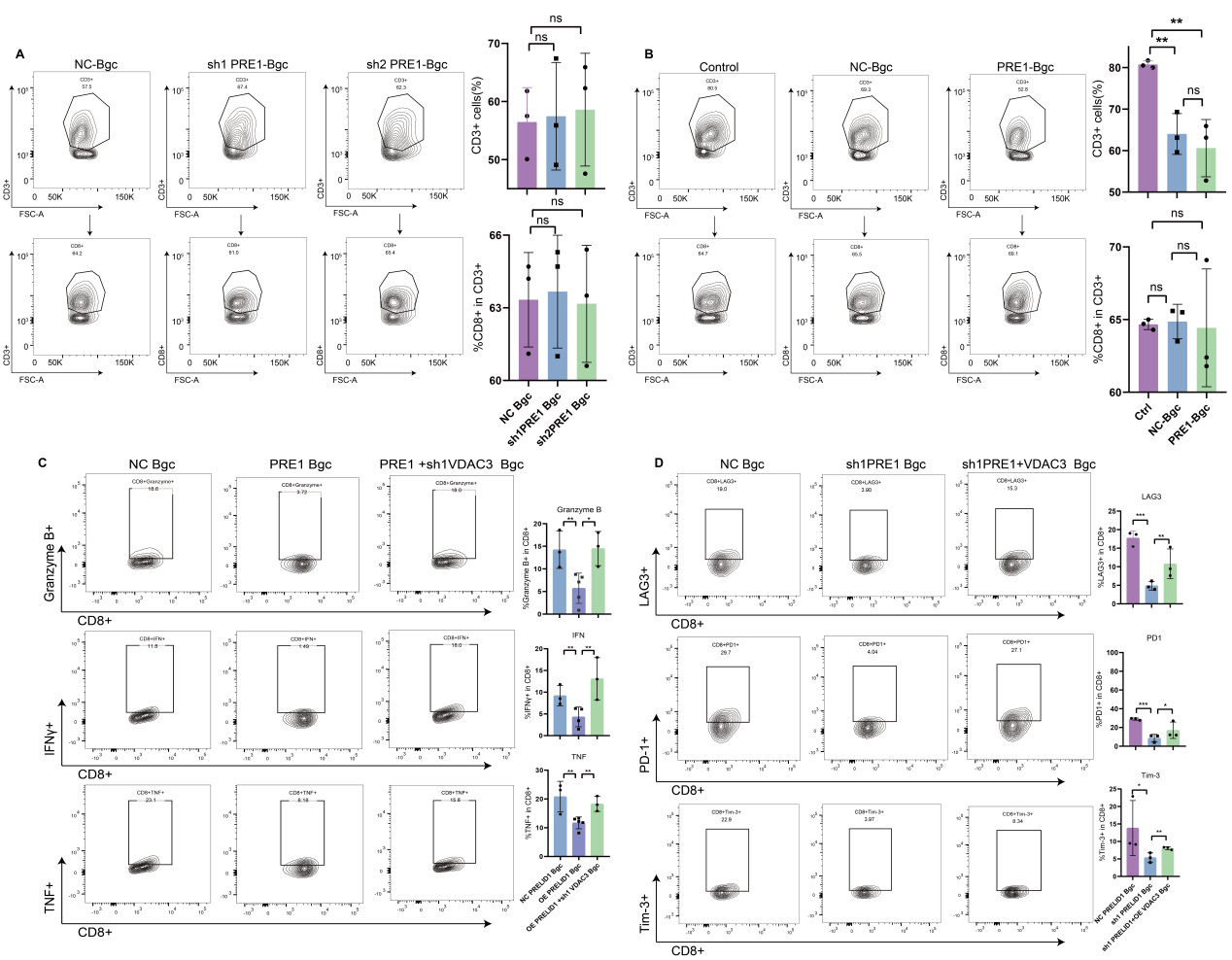
**

**Figure S13. (A)** Representative flow-cytometry plots showing CD3⁺ T-cell proportions. **(B)** Representative flow-cytometry plots showing CD8⁺ T-cell proportions. **(C)** Flow cytometry analysis of function markers (Granzyme B, IFNγ and TNF) on CD8^+^ T cells. **(D)** Flow cytometry analysis of exhaustion markers (PD-1, LAG3, and Tim-3) on CD8^+^ T cells.


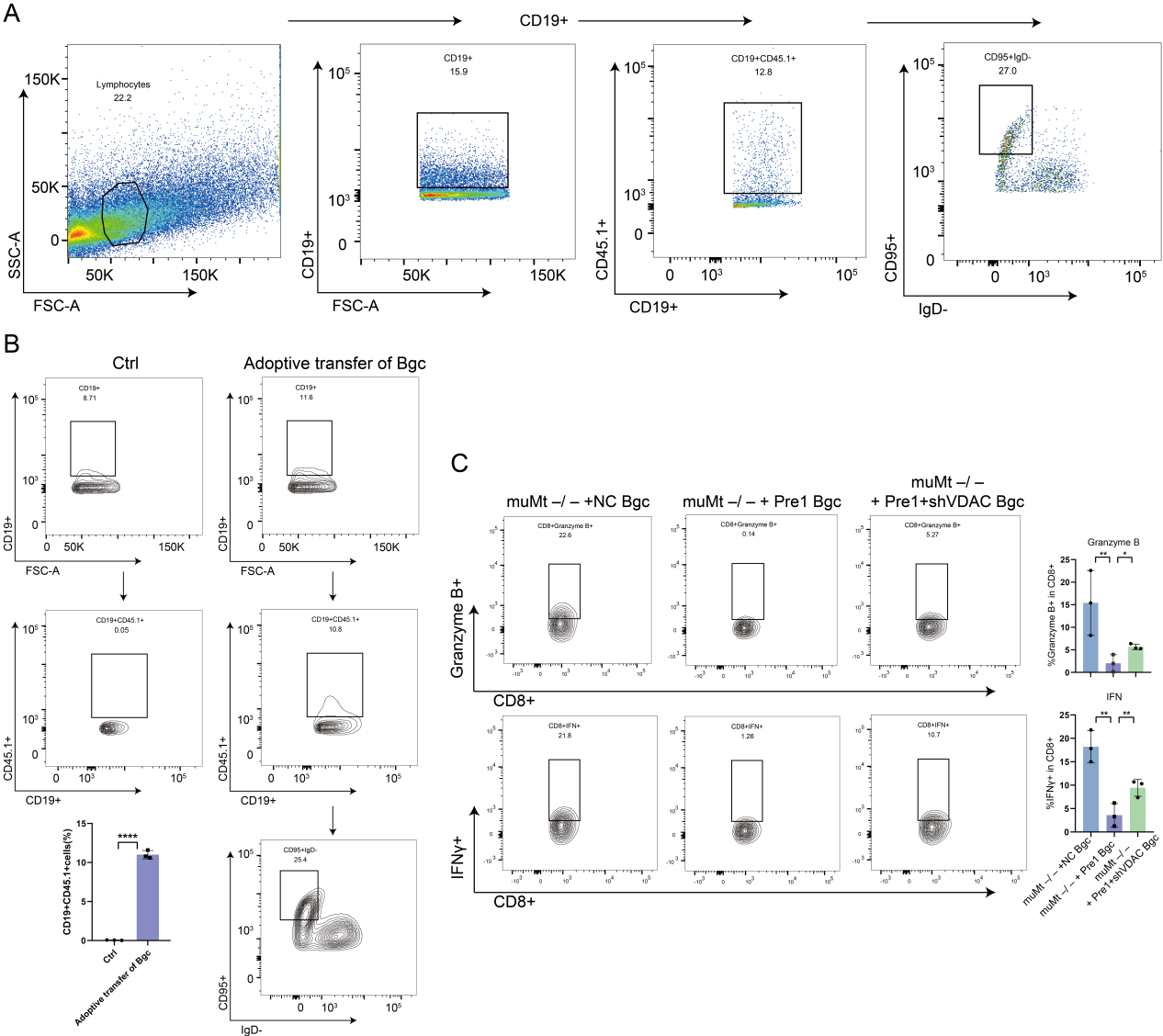


**Figure S14. (A)** Representative flow-cytometric gating strategy used to identify transferred Bgc cells (CD45.1⁺CD19⁺) in tumor tissues. **(B)** Quantification of CD19⁺CD45.1⁺ cells in control and adoptive transfer groups, with CD95⁺IgD⁻ Bgc cells further gated within the adoptive transfer group. Data represent n = 3 mice per group and are presented as mean ± SEM, with statistical comparisons performed using two-sided Student’s t-test. **(C)** Flow cytometry analysis of function markers (Granzyme B, IFNγ and TNF) on CD8^+^ T cells.


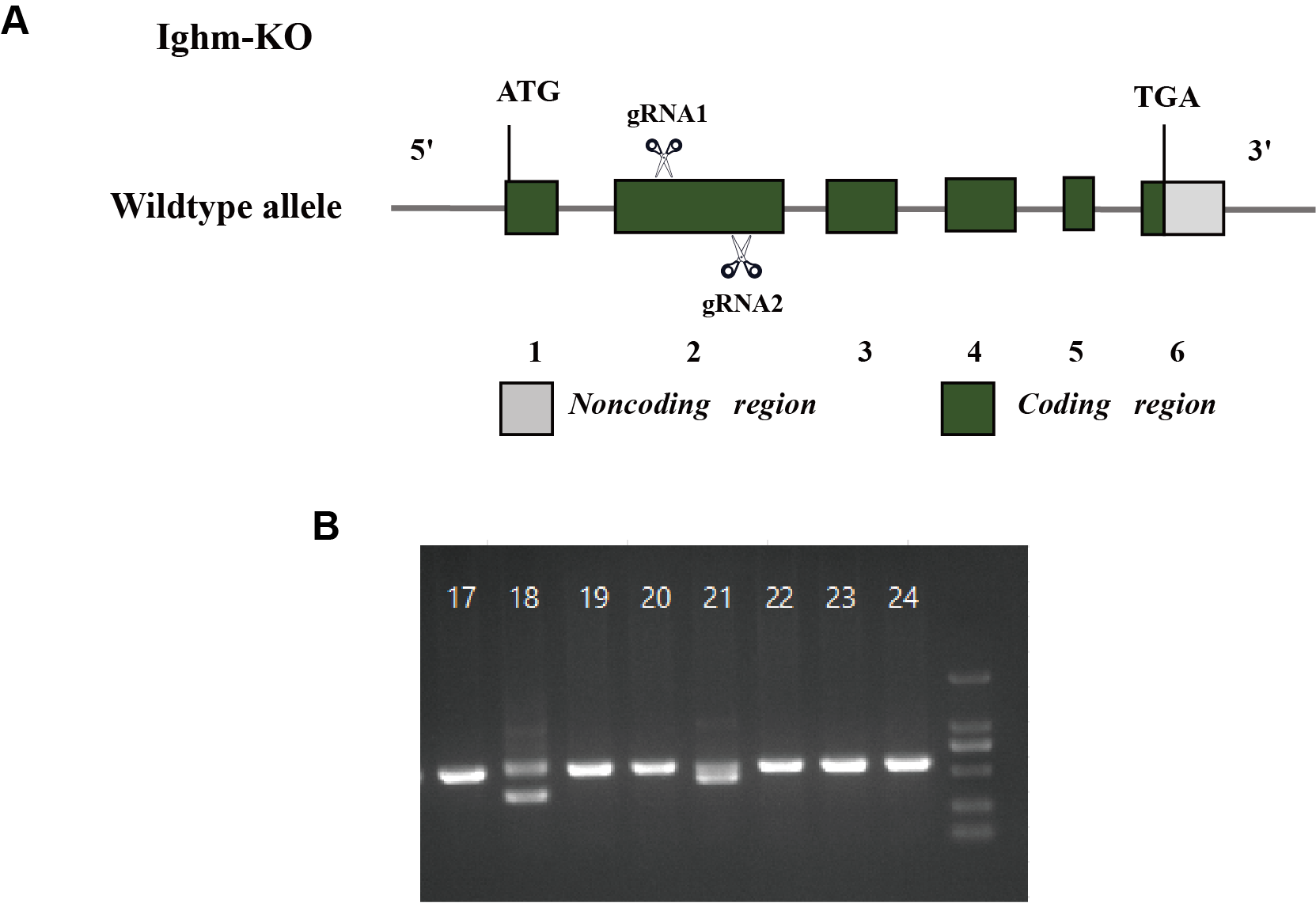


**Figure S15. (A)** Schematic illustration of the transgenic mouse generation strategy. **(B)** Representative PCR electrophoresis results. As shown, all mice were wild-type except for mouse #18, which was heterozygous.


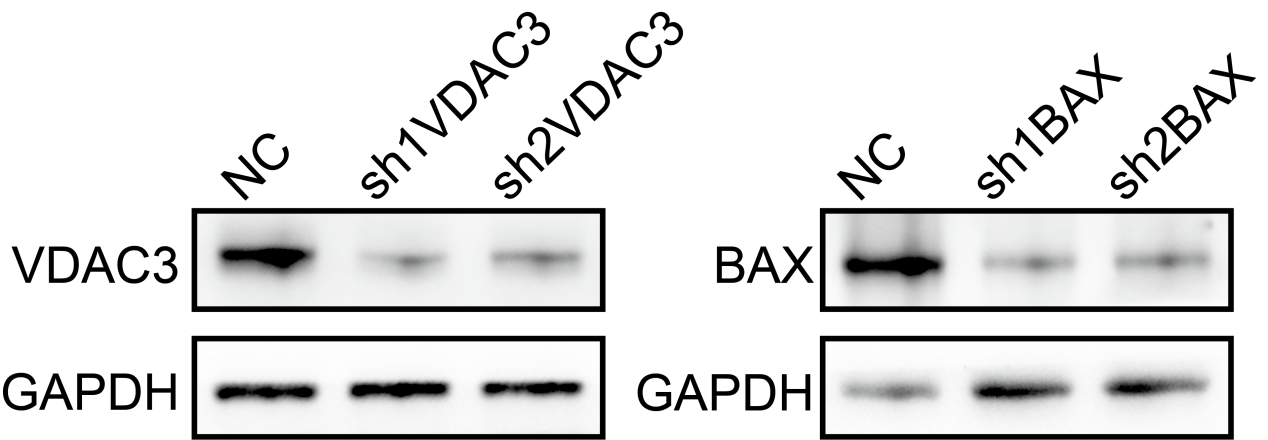


**Figure S16.** Western blot analysis was performed to validate the protein-level knockdown efficiency of shVDAC3 and shBAX.

**Supplementary Table 1. Primers and shRNAs used in this study**

| **Name** | | **Sequence** |
| --- | --- | --- |
| GAPDH (human) | Forward | 5’-AGAAGGCTGGGGCTCATTTG-3’ |
|  | Reverse | 5’-GAGGGGCCATCCACAGTCTTC-3’ |
| PRELID1(human) | Forward | 5’-ACCCACAGAATCAGACCATGAC-3’ |
|  | Reverse | 5’-CAGCCACTGTTGTCAGAGTTC-3’ |
| P16(human) | Forward | 5’-gctgcccaacgcaccgaata-3’ |
|  | Reverse | 5’-ACCACCAGCGTGTCCAGGAA-3’ |
| P21(human) | Forward | 5’-AGGTGGACCTGGAGACTCTCAG-3’ |
|  | Reverse | 5’-TCCTCTTGGAGAAGATCAGCCG-3’ |
| IL7(human) | Forward | 5’-TAAAGACAAGGACGGTAAAGC-3’ |
|  | Reverse | 5’-TTTGGGCAATCACTATCAGTT-3’ |
| shPRELID1(mouse)-1  target sequence | | 5’-CCGGCCCTATAGCAAACATGTCTTACTCGAGTAAGACATGTTTGCTATAGGGTTTTTT-3’ |
| shPRELID1(mouse)-2  target sequence | | 5’-CCGGCCTTGTTGAGACAGCCAAAGACTCGAGTCTTTGGCTGTCTCAACAAGGTTTTTT-3’ |
| shPRELID1(human)-1  target sequence | | 5’-CCGGGCAGCAGCAACAGTTTGTGTACTCGAGTACACAAACTGTTGCTGCTGCTTTTTG-3’ |
| shPRELID1(human)-2  target sequence | | 5’-CCGGGACTACCTTCACCTGGAACATCTCGAGATGTTCCAGGTGAAGGTAGTCTTTTTG-3’ |
| shVDAC3(mouse)-1  target sequence | | 5’-CCGGGCAACCTAGAAACCAAATATACTCGAGTATATTTGGTTTCTAGGTTGCTTTTTG-3’ |
| shVDAC3(mouse)-2  target sequence | | 5’-CCGGCCAGGAGTCAAATTGACTTTACTCGAGTAAAGTCAATTTGACTCCTGGTTTTTG-3’ |
| shVDAC3(human)-1  target sequence | | 5’-CAGTCGACTGGATCCATGTGTAACACACCAACGTACTGTGAC-3’ |
| shVDAC3(human)-2  target sequence | | 5’-TGGGTCTAGATATCTAGCTTCCAGTTCAAATCCCAAGCC-3’ |
| shVDAC2(mouse)-  target sequence | | 5’-CAGTCGACTGGATCCATGAGCTGGTGTAATGAGCTCAGATTGC-3’ |
| shVDAC2(mouse)-  target sequence | | 5’-TGGGTCTAGATATCTAGCCTCCAACTCCAGGGCGAG-3’ |
| shVDAC1(mouse)-  target sequence | | 5’-AAAAGGATCCATGGCTGTGCCACCCACGTA-3’ |
| shVDAC1(mouse)-  target sequence | | 5’-ATCTTGCTTGAAATTCCAGTCCTAGACCAAGCT-3’ |
| shTRPML1(human)-1  target sequence | | 5’-TCAGTCGACTGGATCCCTACAGCCCCGGCGGGTCC-3’ |
| shTRPML1(human)-2  target sequence | | 5’-ATCTCGAGTGCGGCCGCTCAATTCACCAGCAGCGA-3’ |
| shTRPML1(mouse)-1  target sequence | | 5’-ACAAAGTTGACTGTGAAGCTGTAC-3’ |
| shTRPML1(mouse)-2  target sequence | | 5’-GTTCATCAGCTGGGCACCTA-3’ |
| lghm-KO-IF | | 5’-TGTGCCCATTCCAGGTAAG-3’ |
| lghm-KO-IR | | 5’-CCAAAGTTCAAGGAGCAAATG-3’ |

**Supplementary Table 2. Antibodies used in this study**

| **Antibodies** | **SOURCE** | **CATALOG** |
| --- | --- | --- |
| Alexa Fluor® 700 anti-mouse CD45.1 | Biolegend | 103127 |
| Alexa Fluor® 700anti-mouse CD45.2 | Biolegend | 560693 |
| Brilliant Violet 605™ anti-mouse CD8a | Biolegend | 100743 |
| FITC anti-mouse CD8a | Biolegend | 100705 |
| APC anti-mouse IFN-γ | Biolegend | 505809 |
| Brilliant Violet 421™ anti-mouse TNF-α | Biolegend | 506327 |
| Alexa Fluor® 700 anti-human/mouse Granzyme B | Biolegend | 372221 |
| Brilliant Violet 605™ anti-mouse CD279 (PD-1) | Biolegend | 135219 |
| Brilliant Violet 510™ anti-human CD223 (LAG-3) | Biolegend | 369317 |
| PerCP/Cyanine5.5 anti-mouse CD366 (Tim-3) | Biolegend | 134011 |
| APC/Fire™ 750 anti-mouse CD3 Antibody | Biolegend | 100247 |
| PE anti-mouse CD38 | Biolegend | 102707 |
| APC anti-mouse CD95 (Fas) | Biolegend | 152603 |
| PE/Cyanine7 anti-mouse CD95 (Fas) | Biolegend | 152617 |
| Pacific Blue™ anti-MU/HU GL7 Antigen (T/B Cell Act. Marker) | Biolegend | 144613 |
| Brilliant Violet 605™ anti-mouse IgD | Biolegend | 405727 |
| anti-GAPDH | Cell Signaling Technology | 2118 |
| anti-TOM20 | Santa Cruz Biotechnology | sc-11415 |
| anti-UQCRC1 | Invitrogen | 459140 |
| Anti-CD8 alpha antibody | Abcam | ab4055 |
| anti-pUb | Cell Signaling Technology | 62802 |
| anti-PINK1 | Invitrogen | PA5-86941 |
| anti-Gal3 | Santa Cruz Biotechnology | sc-23938 |
| anti-CHMP4B | Proteintech | 13683-1-AP |
| anti-ALIX | Abcam | ab76608 |
| anti-VPS4 | Sigma | SAB4200025 |
| anti-GFP | Cell Signaling Technology | 2555 |
| anti-LAMP1 | Santa Cruz Biotechnology | sc-20011 |
| Anti-p21 antibody [EPR362] | Abcam | ab109520 |
| Anti-CDKN2A/p16INK4a antibody [EPR24167-43] | Abcam | ab270058 |
| Anti-CD20 | Abcam | ab64088 |
| Anti-PRELID1 | Invitrogen | PA5-31087 |

**References**

1. Jin, S. et al. Inference and analysis of cell-cell communication using CellChat. *Nat. Commun.* **12**, 1088 (2021).

2. Subramanian, A. et al. Gene set enrichment analysis: A knowledge-based approach for interpreting genome-wide expression profiles. *Proceedings of the National Academy of Sciences*. **102**, 15545-15550 (2005).

3. Wu, T. et al. clusterProfiler 4.0: A universal enrichment tool for interpreting omics data. *Innovation-Amsterdam*. **2**, (2021).

4. Liao, Y. et al. N6-methyladenosine RNA modified BAIAP2L2 facilitates extracellular vesicles-mediated chemoresistance transmission in gastric cancer. *J. Transl. Med.* **23**, 320 (2025).
